# Unique bone histology of modern giant salamanders: a study on humeri and femora of *Andrias spp.*

**DOI:** 10.1186/s40851-024-00240-1

**Published:** 2024-10-18

**Authors:** Nicole Klein, Dorota Konietzko-Meier, Sudipta Kalita, Masahiro Noda, Sena Ishikawa, Yuki Taguchi, Wataru Anzai, Shoji Hayashi

**Affiliations:** 1 Bonn Institute of Organismic Biology (BIOB-V), Section Paleontology, University of Bonn, Nussallee 8, Bonn, 53115 Germany; 2 Stuttgart State Museum of Natural History, Rosenstein 1, 70191 Stuttgart, Germany; 3 Department of Biology, University of Dayton, 300 College Park Dayton, Dayton, OH 45469 USA; 4 Department of Biosphere-Geosphere Science, Okayama University of Science, Ridai-Cho 1-1, Kita-Ku, Okayama, 700005 Japan; 5 Graduate School of Human and Environmental Studies, Kyoto University, Yoshida Nihonmatsu-Cho, Sakyo-ku, Kyoto, 6068501 Japan; 6 Hiroshima City Asa Zoological Park, Asa-cho, Asakita-ku, Hiroshima City, Hiroshima, 7313355 Japan; 7 Asahi Hanzaki Research Association, Asahigaoka 2-14-31, Asakita-Ku, Hiroshima City, Hiroshima, 7313361 Japan; 8 Division of Materials and Manufacturing Science, Graduate School of Engineering, Osaka University, 2-1, Yamada-Oka, Suita, Osaka 565-0871 Japan

**Keywords:** Petrographic thin sections, Coarse parallel-fibred tissue, Avascular tissue, Osteocyte lacunae size, Canaliculi, Sharpey’s fibers, Growth marks

## Abstract

The osteohistology of *Andrias* spp. is a pivotal analogue for large fossil non-amniotes (e.g., Temnospondyli), and the endangered status of this taxon underlines the importance of gathering information on its growth. We here present the first osteohistological study by petrographic thin sections of an ontogenetic series of humeri and femora of eight individuals of varying sizes (28.5–104 cm) and ages (2.5–32 years) of *Andrias japonicus* from the Hiroshima City Asa Zoological Park, Japan. In addition, two individuals of *A.* cf. *davidianus* of unknown age but of different size (62 cm and 94 cm) were studied. All samples of *Andrias* spp. show a primary avascular periosteal cortex made of parallel-fibred tissue around the ossification center in the petrographic thin sections. Mainly in small individuals, the fibers forming this tissue are very coarse and loosely organized. With increasing size and age, the coarse tissue is irregularly intermixed and later replaced with finer and better organized fibers. This histologic change is accompanied by a change from diffuse annuli in the inner cortex to distinct lines of arrested growth (LAGs) in the outer cortex. We interpret these changes in tissue and the appearance of distinct growth marks as indicating the onset of active reproduction. The lack of primary vascularization around the ossification center in our *Andrias* spp. sample is striking and contradicts other observations. Vascularity may be prone to plasticity and further studies are necessary. We hypothesize that the large osteocyte lacunae and the dense networks of canaliculi observed in our sample may have nourished the tissue instead of primary vascular canals. We measured the size of osteocyte lacunae of *Andrias* spp. in comparison to other Lissamphibia, and found them to be significantly larger throughout ontogeny. The periosteal cortex contains a high amount of thick Sharpey’s fibers all around the midshaft cross sections. The two samples of *Andrias* cf. *davidianus* show tissue and growth mark distribution similar to that observed in *A. japonicus*. However, the large individual of *A.* cf. *davidianus* differed in its extremely osteosclerotic condition and the retention of a small layer of calcified cartilage in the endosteal region of the femur. It remains unclear whether these differences are related to plasticity, taxonomy, sex, exogenous factors, or attributable to a regenerated but fully regrown leg. Although the present study is based on zoo-kept and not wild, animals, it yields important insights into osteohistological plasticity and growth patterns in giant salamanders.

**Supplementary Information:**

The online version contains supplementary material available at 10.1186/s40851-024-00240-1.

## Background

Giant salamanders are characterized by their unusually large body size and long lifespan compared to other lissamphibians. These features and their slow growth, low metabolic rate, delayed maturity, and paedomorphosis make them fascinating animals to study [52]. However, these life history traits in combination with habitat loss renders aquatic giant salamanders an endangered species, identifying them as critical conservation targets [78, 21]. In China pure wild populations are highly endangered or already extinct [53, 74]. In Japan, research on wild populations is not allowed without special permission from Japanese government, as *Andrias japonicus* is designated as a “special natural monument.” Thus, detailed knowledge on their biology and life history strategy remains sparse, and studies must rely on zoo animals.

The fossil record of the genus *Andrias* dates back to the Oligocene of Europe (e.g., [1, 79]). The first fossil of a giant salamander was found in 1725 in southern Germany and became famous as “*Homo diluvii testis*”, resulting from a misinterpretation of the skeleton as a human witness of the biblical Deluge [61]. This was later corrected, and the fossil taxon *Andrias scheuchzeri* [28, 70] was erected. Today, *Andrias* is restricted to East Asia and it is referred to as a “living fossil” as its morphology has remained unchanged since the Oligocene.

*Andrias* belongs to the Cryptobranchidae, a primitive lineage of lissamphibians that dates back to the Jurassic period [22]. There are two known extant genera of giant salamanders [10]. The hellbender *Cryptobranchus alleganiensis* lives in the United States, and reaches a body length of up to 74 cm [19]. Five species are recognized within the modern genus *Andrias*: *A. japonicus* from Japan; and *A. davidianus*, *A. cheni, A. jiangxiensis* and *A. sligoi* from China [2, 10]. *Andrias japonicus* can grow up to 143 cm with a weight of 27.6 kg [45]. Kawakami et al. [33] reported an individual weighing 44.3 kg. *A. davidianus* can reach 180 cm [9]. The genus *Andrias* exhibits also a remarkable longevity with over 50 years of breeding records [34, 76].

*Andrias* spp. are aquatic, spending their entire lives under water. They undergo a partial metamorphosis from an aquatic larval stage to aquatic adults with a concomitant transfer of respiration from external gills to lungs [23]. The hyobranchial apparatus of the adult retains some larval features, for which reason they are regarded as neotenic [15, 16]. Metamorphosis, i.e., resorption of the external gills, is documented in the second year of life in *Andrias davidianus* [14] and occurs around the third year of life in *A. japonicus* [17, 25]. According to Suzuki [68], in captive individuals at the Hiroshima City Asa Zoological Park (hereafter abbreviated as Asa Zoo), the external gills were almost completely lost after three years and the gill slits are closed after the fourth year of life.

In the wild, *Andrias japonicus* inhabits mountain streams, and during the breeding season males and females migrate mainly upstream to nest in dens [77]. These dens are often guarded by a dominant large male, known as the den master, who mates with the accepted females that additionally mate with several subordinate males [47, 71]. In previous studies, the smallest males and females that participated in the spawning behavior measured 26.7–32.2 cm and 39–44.5 cm respectively, suggesting sexual maturity is attained at these sizes in wild populations [47, 43, 44]. The exact age at which sexual maturity starts in wild populations remains unknown. Long-term observations in the Asa Zoo documented a body size of about 73.5 cm and an age of 15 years when captive females spawn [27]. The size at which active reproduction starts in captive females is thus larger when compared to those in the wild. That growth rates vary between captive and wild individuals is well documented [73, 27] and is usually interpreted as being related to a higher available amount of food for captive individuals, resulting in a faster growth. Tochimoto [73] measured a growth rate of 5 mm per year in a wild population at Hyogo and, Kobara [44] measured 6 mm per year in a wild population near Hiroshima. Kuwabara et al. [46] found the onset of active reproduction in *Andrias* was more strongly related to size than to reaching a certain age. However, conversely, individuals that are well fed quickly attain a large size but do not begin active reproduction earlier [27]. There is no visible external sign of sexual dimorphism, except for swollen lips of the cloaca in males during the breeding season. However, poor nutrition may prevent the swelling of cloacal lips during the breeding season [73].

Overall, there is a consensus that *Andrias* spp. exhibits strong developmental plasticity and that body size is a poor indicator for estimating the age of individuals (e.g., [11, 26, 35]). Understanding the growth pattern, age structure, and age at which active reproduction starts is vital for the conservation of wild populations. However, acquiring such information has remained a challenge, and an accurate age determination method for *Andrias* needs to be established. Thus, any reliable new information on their growth rate and patterns is important.

## Aim

This study describes the osteohistology in petrographic thin sections of humeri and femora of *Andrias japonicus* from the Hiroshima City Asa Zoological Park. Individuals are of known age and size. Additionally, two different-sized zoo-kept individuals of unknown age of *A.* cf. *davidianus* were studied to complement the primary observations.

The aim of the present study is to gain a better understanding of growth at the tissue level, and to contribute to our knowledge about general growth patterns in these giant salamanders. These insights may help to understand life history traits and growth strategies in *Andrias* spp. Osteohistological and growth data of *Andrias* spp. will also substantially assist in interpreting osteohistological data from large extinct non-amniotes, such as Temnospondyli. For any extinct vertebrate, osteohistology is the only method for obtaining direct data on life history traits.

The present study represents the initial phase of ongoing research designed to elucidate the growth patterns and life history of *Andrias* spp., which will aid the understanding of the biology of wild populations and contribute to the design of effective conservation strategies.

## Previous studies on microanatomy and bone tissue of *Andrias* spp.

Limb elements of *Andrias japonicus* were included in microanatomical analyses of Laurin et al. [49–51], which revealed osteosclerotic conditions but reported no further histological information. Sanchez et al. [60:suppl.Fig. 7] studied a humerus of *Andrias* sp. by phase-contrast X-ray synchrotron radiation microtomography, finding a large spongy medullary region, radial oriented simple vascular canals, and numerous small osteocyte lacunae [60].

A detailed histological description of petrographic longitudinal and transverse sections of humeri and femora of *Andrias japonicus* and *Andrias davidianus* is given by Canoville et al. [12]. The femora of both taxa show a few simple vascular canals, whereas the humeri are avascular. The tissue is described as parallel-fibred with poor birefringence, poorly developed canaliculi, and “osteocyte lacunae being predominantly circular in transversal sections, with some variability in their morphology and orientation” [12:114]. Lines of arrested growth (LAGs) are clearly visible and both humeri and femora exhibit large erosion cavities with sparse secondary redeposition [12]. The presence of calcified cartilage at the adult stage in the medullary cavities of *Andrias* spp. is interpreted as evidence of neoteny [12].

The vascular network of a femur of *Andrias* sp. was 3D reconstructed by micro-Ct-data by Buffrénil et al. [6]. In Buffrénil et al. [7] a femur and humerus of *A. japonicus* are shown which is very similar in tissue composition to what Canoville et al. [12] described for *A*. *davidianus*. However, contrary to Canoville et al. [12], Buffrénil et al. [7] describe the femur of *A. japonicus* as avascular. Buffrénil and Laurin [3] compared the *Andrias* sample of Canoville et al. [12] with that of Sanchez et al. [60] and pointed out that the differences in vascular density may be the result of a substantial inter-individual variability. Buffrénil et al. [8] depict a femoral sample of *A. japonicus* that shows few simple vascular canals. All samples of *Andrias* spp. in Buffrénil et al. [6–8] seem to refer to the same specimens as those of Canoville et al. [12] and indicate room for interpretation.

Yamasaki et al. [80] conducted a study on the skeletochronology of *Andrias japonicus,* analyzing microtomic thin sections from the phalanges of specimens that lived and died in captivity at Asa Zoo. The study included individuals ranging from 7.8 cm (1 year old at death) to 55 cm (11 years old at death). They found that the number of lines of arrested growth (LAGs) was consistently one less than the number of winters each individual had lived.

## Material

### *Andrias japonicus*

Since 1979, Asa Zoo has been breeding and rearing Japanese giant salamanders, providing access to specimens at various growth stages with known ages [38, 46]. This historical depth makes it possible to use these specimens to study the relationship between age and histology, potentially leading to the development of reliable age determination methods for the genus *Andrias*. Eight individuals of *Andrias japonicus* from the Asa Zoo were sampled by SH, MN, and SI. The specimens hatched and died in the Asa Zoo, and were maintained in life by YT, WA, and colleagues. The conservation breeding center of the Japanese giant salamander in the Asa Zoo is an outside facility, resembling natural conditions (i.e., light and temperature) as closely as possible. Individuals were housed in groups in containers. For breeding, well and stream water were used, but only well water was used for rearing juvenile and keeping adult salamanders. *A. japonicus* hatch in the Asa Zoo usually in mid to late October. The age of individuals is calculated based on a fictive birth date of October 15th and the day of death. Water temperature ranges over the year from 10 to 21°C. All individuals experienced low water temperature during the winter, especially in February.

Young individuals were fed bloodworms and krill, while adults were fed fish such as horse mackerel, Atka mackerel, and living loach. Individuals were not fed individually but a certain amount of food is added to each tank, generally once a week. Thus, it is not guaranteed that all individuals receive the same amount of food. Some individuals may be less successful in getting food than others. For this reason, growth rates and sizes vary within the same age group, even in animals kept in the same tank [73, 27].

Sometimes individuals die due to being attacked by other individuals. After death, specimens are immediately collected and dissected to, among other reasons, determine sex. Males have confirmed testes visible on ultrasonography at an age of six years and a body length of 36 cm. In females, ovaries are observed by laparoscope at an age of seven years with 50 cm body length [55]. However, the presence of sex organs does not necessarily indicate sexual maturity. Following post-mortem examination, animals are frozen or stored in 10% formalin. All here included individuals show bite marks over their bodies and thus likely died as a result of being attacked.

Although *Andrias* individuals can regenerate their limbs, none of the *A. japonicus* bones included in the present study was a regenerated element. Regenerated limbs can be identified because they are usually considerably smaller than their contralateral partner, and the entire bone tissue is composed of cartilage (pers. obs. SH, SI). Specimens from Asa Zoo are abbreviated as ASAGS. For more information on the individuals included into this study see Table 1.

**Table 1:** Material of *Andrias japonicus *(ASAGS) and* A. davidianus* (ZFMK) ordered after age (ASAGS sample) and size (ZFMK sample)

**repository number**	**age, sex, body length**	**Characters**	**Humerus **	**Femur **
**ASAGS-0078**	2 yrs., 4 mths; unknown sex; 305 mm	**sampling location**	8.09 mm; sampled at ossification center	10.3 mm; sampled at ossification center
		**medullary cavity**	29%	13%
		**closed layer of endosteal bone around medullary cavity**	present	present
		**Kastschenko’s line**	present	present
		**resorption**	absent	absent
		**periosteal cortex**	avascular coarse parallel-fibred tissue; inner layer of highly organized parallel-fibred tissue; dense network of canaliculi; deep scares by Sharpey’s fibers	
		**growth marks**	diffuse annuli	
**ASAGS-0077**	3 yrs., 4 mths; ?female; 360 mm	**sampling location**	11.52 mm; sampled at ossification center	13.07 mm; sampled at ossification center
		**medullary cavity**	~40%	28%
		**closed layer of endosteal bone around medullary cavity**	damaged inner cortex	present
		**Kastschenko’s line**	present	absent
		**resorption**	damaged inner cortex	started at margin of medullary cavity
		**periosteal cortex**	avascular, very coarse parallel-fibred tissue; inner layer of highly organized parallel-fibred tissue only in femur visible due to damage in humerus; thick numerous osteocyte lacunae; dense network of canaliculi; short and long, thick fibers, Sharpey’s fibers	
		**growth marks**	diffuse annuli	
**ASAGS-0050**	8yrs, 6 mths; female; 285 mm	**sampling location**	10.39 mm; sampled at ossification center; nutrient canal as foramen	9.88 mm; sampled off ossification center
		**medullary cavity**	17% filled by chondrocytes & cc	53%
		**closed layer of endosteal bone around medullary cavity**	present	present
		**Kastschenko’s line**	absent	absent
		**resorption**	absent	started at margin of medullary cavity
		**periosteal cortex**	avascular, coarse parallel-fibred tissue; locally thin inner layer of highly organized parallel-fibred tissue in humerus; osteocyte lacunae flat; dense network of canaliculi; Sharpey’s fibers & short fibers numerous except for dorsal bone side	
		**growth marks**	diffuse annuli	
**ASAGS-0317**	6 yrs., 7 mths; unknown sex; 425 mm	**sampling location**	*humerus not sampled*	17.29 mm; sampled at ossification center
		**medullary cavity**	na	39%
		**closed layer of endosteal bone around medullary cavity**	na	present but incomplete
		**Kastschenko’s line**	na	absent
		**resorption**	na	started at margin of medullary cavity
		**periosteal cortex**	avascular, highly organized parallel-fibred tissue; colorless osteocyte lacunae; no canaliculi visible; Sharpey’s fibers all around	
		**growth marks**	na	diffuse annuli
		**outer cortex**	na	initially wave-like
**ASAGS-0100**	13yrs.,10mths; female; 735 mm	**sampling location**	29.78 mm; sampled at ossification center; nutrient canal	31.81 mm; sampled at ossification center; nutrient canal
		**medullary cavity**	16%	50%
		**closed layer of endosteal bone around medullary cavity**	interrupted by erosion	interrupted by erosion
		**Kastschenko’s line**	absent	absent
		**resorption**	present in form of erosion cavities and some secondary endosteal deposits	present in form of erosion cavities and intensive secondary endosteal deposits
		**periosteal cortex**	avascular, highly organized parallel-fibred tissue; inner tissue differently (less high) organized in humerus; osteocyte lacunae colorless; canaliculi locally accumulated; Sharpey’s fibers mainly ventrally and anteriorly	
		**growth marks**	diffuse annuli in inner cortex and LAGs in outermost cortex	
		**outer surface**	initially wave-like	wave-like
**ASAGS-0197**	18yrs.,11 mths male; 700 mm	**sampling location**	30.47 mm; sampled off ossification center	31.7 mm; sampled off ossification center
		**medullary cavity**	60% alcified cartilage in the medullary region	54%
		**closed layer of endosteal bone around medullary cavity**	interrupted by erosion	interrupted by erosion but still nearly closed
		**Kastschenko’s line**	absent	absent
		**resorption**	intensive, present in form of erosion cavities and some secondary endosteal deposits	present in form of erosion cavities and some secondary endosteal deposits
		**periosteal cortex**	avascular, very coarse parallel-fibred tissue; partially highly organized parallel-fibred tissue; large osteocyte lacunae; dense network of canaliculi; thick Sharpey’s fibers mainly ventroanteriorly	
		**growth marks**	gm mainly by an alignment of osteocyte lacunae, become clearer/more distinct in outer cortex	
		**outer surface**	initially wave-like	wave-like
**ASAGS-0106**	24yrs,10 mths; female; 775 mm	**sampling location**	30.57 mm; sampled at ossification center	34.93 mm; sampled off ossification center; nutrient canal
		**medullary cavity**	23%	70%
		**closed layer of endosteal bone around medullary cavity**	present (at one place interrupted)	interrupted by erosion
		**Kastschenko’s line**	absent	absent
		**resorption**	only locally	intensive
		**periosteal cortex**	avascular, highly organized parallel-fibred tissue; colorless osteocyte lacunae, canaliculi not visible; moderate/locally Sharpey’s fibers	
		**growth marks**	diffuse annuli in inner cortex; closely spaced LAGs outer cortex	
		**outer surface**	initially wave-like	wave-like
**ASAGS-0101**	32yrs, 8 months; female; 1040 mm	**sampling location**	45.769 mm; sampled at ossification center	45.77 mm; sampled at ossification center
		**medullary cavity**	23%	20%
		**closed layer of endosteal bone around medullary cavity**	interrupted by erosion and remodeling	interrupted by erosion and remodeling
		**Kastschenko’s line**	absent	absent
		**resorption**	intensive erosion and some secondary endosteal bone deposits	intensive erosion and some secondary endosteal bone deposits
		**periosteal cortex**	highly organized avascular parallel-fibred tissue; partially colorless osteocyte lacunae, canaliculi locally accumulated; Sharpey’s fibers locally all around	
		**growth marks**	diffuse annuli in inner half of cortex and LAGs in outer half of cortex	
		**outer surface**	initially wave-like	wave-like
**ZFMK 8568**	unknown age; female; 620 mm	**sampling location**	29 mm; a bit off ossification center; nutrient canal as foramen	27.6 mm; sampled ossification center; nutrient canal as foramina
		**medullary cavity**	11%	44%
		**closed layer of endosteal bone around medullary cavity**	interrupted by erosion	interrupted by erosion
		**Kastschenko’s line**	absent	absent
		**resorption**	intensive erosion	intensive erosion
		**periosteal cortex**	avascular, partially coarse and loose, partially highly organized parallel-fibred tissue; osteocyte lacunae are small but numerous; dense network of canaliculi; Sharpey’s fibers locally all around	
		**growth marks**	diffuse annuli	
		**outer surface**	smooth	initially wave-like
**ZFMK 97391**	unknown age; female; 940 mm	**sampling location**	45 mm; sampled at ossification center; some aligned openings might indicate remains of the nutrient canal	45.5 mm; sampled at ossification center
		**medullary cavity**	14.5%	5%; medullary cavity surrounded by alcified cartilage
		**closed layer of endosteal bone around medullary cavity**	present	present
		**Kastschenko’s line**	absent	absent
		**resorption**	absent	absent
		**periosteal cortex**	avascular highly organized parallel-fibred tissue; more loosely organized in the femur; higher organized early ontogenetic stage in inner cortex; tiny osteocyte lacunae; crushed network of tiny canaliculi; Sharpey’s fibers locally all around	
		**growth marks**	diffuse annuli in inner cortex, LAGs in outer cortex	
		**outer surface**	initially wave-like	wave-like

All samples were handled according to the laws and regulations of the Agency for Cultural Affairs, Japan.

### *Andrias* cf. *davidianus*

The left humerus and right femur of two individuals that were assigned to *Andrias* cf. *davidianus* (ZFMK 8568, ZFMK 97391) had been sampled first hand by NK and DKM at Zoologisches Forschungsmuseum Alexander Koenig (hereafter ZFMK). ZFMK 8568 had been mentioned by Canoville et al. [12] and Buffrénil et al. [6–8]. ZFMK 97391 may correspond to the specimen of *A*. cf. *davidianus* labelled as “ZFMK unnumbered” in Canoville et al. [12] and Buffrénil et al. [3, 5–8]. Both individuals lacked their right humerus and left femur when sampled by us. According to the documents of the ZFMK, those bones had been taken for histological sampling by French colleagues. Both specimens had been stored in ethanol. ZFMK 97391 was a captive-kept animal but nothing is known about its age or housing conditions. ZFMK 8568 was born in wilderness and ended life in captivity. Again, no information about housing conditions or age and size at time of capture are available. ZFMK 97391 was a female with a total body length of 94 cm. ZFMK 8568 was a female and has a total length of 62 cm. All limb elements of the ZFMK samples looked equally in size and thus, none was an obviously regenerated one.

Note that the current sample of *Andrias* spp. shows a bias towards female.

## Methods

Before sampling, micro-Ct-scans of the ZFMK specimens were performed. ZFMK 97391 was scanned with a v|tome|x s scanner manufactured by GE phoenix|X-ray (Wunstorf, Germany). The micro-Ct machine is operated by the Bonn Institute of Organismic Biology, Section Paleontology, at the University of Bonn (Bonn, Germany). ZFMK 8568 was scanned with the Skyscan 1272 at the ZFMK.

All bones from the Asa Zoo were scanned using an experimental animal X-ray Ct scanner (Latheta LCT-200, Aloka; 24-μm resolution, 80 kV, 0.2 mA) at Okayama University of Science (Okayama, Japan).

**Fig. 1:**
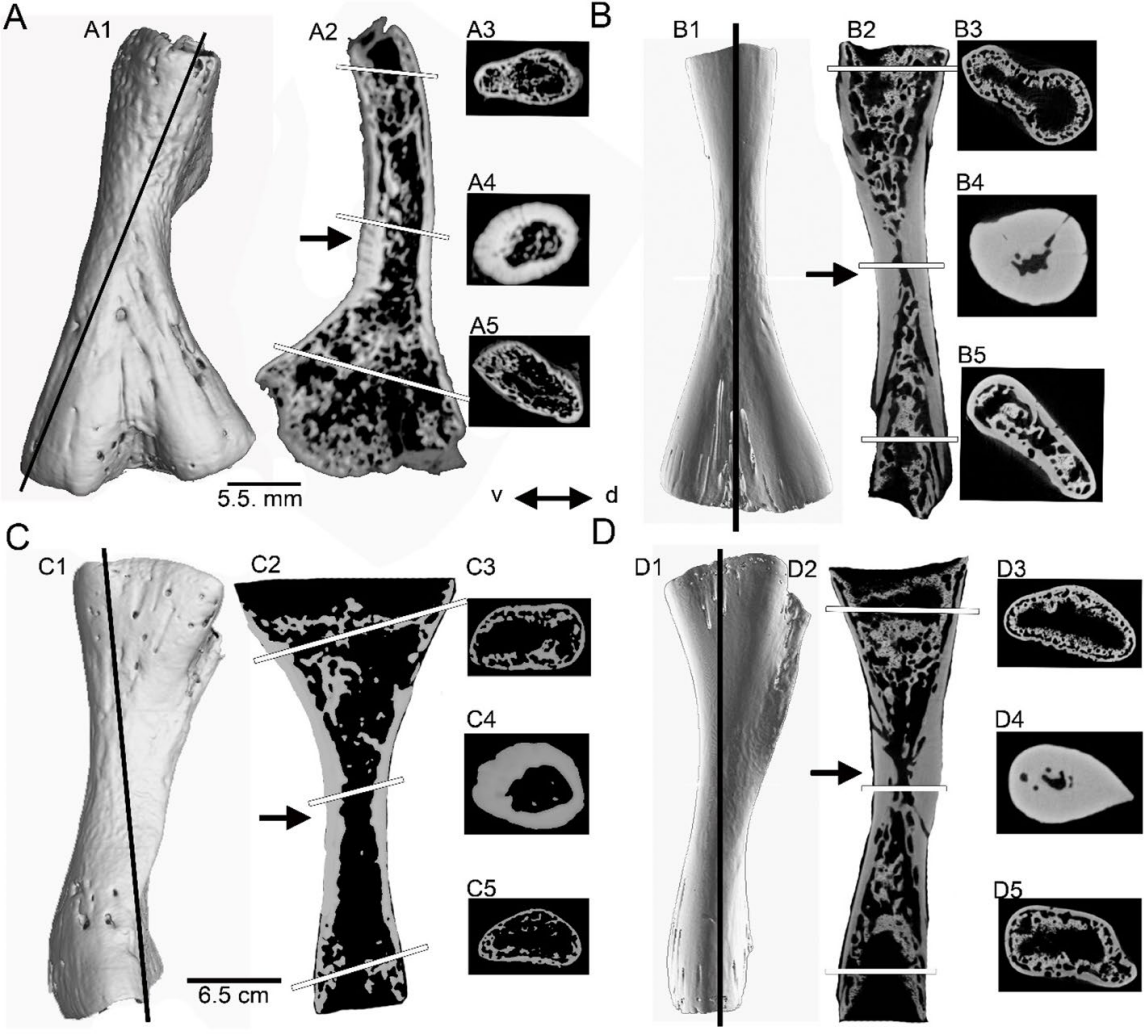
Exemplary sampling location in the humeri and femora of *Andrias* spp. based on micro-Ct-photographs. **A**, humerus of *Andrias japonicus* (ASAGS-0197). **B**, Humerus of *Andrias *cf. *davidianus *(ZFKM 8568). **C**, Femur of *Andrias japonicus *(ASAGS-0197). **D**, Femur of *Andrias *cf. *davidianus *(ZFKM 8568). **A1**-**D1**, micro-Ct-photographs of the respective bone in posterior view. **A2**-**D2** micro-Ct-photographs of section through the longitudinal axis of the respective bone. Please note that the longitudinal sections in *Andrias japonicus *are not exactly longitudinal but angled (**A2**,** C2**) because the entire forelimb was scanned and the humerus was not aligned perpendicular to its long axis (i.e., not parallel to the vertical rotation axis of the x-ray). In *Andrias *cf. *davidianus* the section plane shown in **B2** and **D2** is the longitudinal view of the respective bone. The bright areas in the micro-Ct-photographs represent bone and the black areas indicate cavities. The ossification center is identified as the area where the cortex is thickest (arrows in **A2**-**D2**). Towards the proximal and distal ends, the cortex thins out. **A3**-**D3**, micro-Ct-photographs of the cross section through the proximal head. Note the high porosity of the endosteal region traversed by trabecles and surrounded by a thin periosteal cortex. **A4-D4**, micro-Ct-photographs of cross section through the ossification center. Note the relatively thick and avascular periosteal cortex. Humerus ZFKM 8568 shows at the ossification center the nutrient canal (**B4**). The nutrient canal is not visible at the ossification center in the femur of the same individual or in ASAGS-0197. **A5-D5**, micro-Ct-photographs of the cross section through the distal end. Note the high porosity of the endosteal region traversed by trabecles and surrounded by a thin periosteal cortex. Please note that the periosteal cortex does not show any cavities. Cavities are restricted to the endosteal region that is surrounded by the periosteal cortex. This observation is confirmed by the petrographic thin sections from the area of the ossification center. The black bars in **A1-D1** indicate the section plane shown in **A2-D2**; the white bars in **A2-D2** indicate the section plane of the cross sections shown in **A3-D5**. Abbreviations: d, dorsal; v, ventral

Micro-Ct-data were used to identify the region along the shaft where the growth record is most completely preserved, indicated by the area with the thickest cortex and the smallest medullary region and/or cavity (Fig. 1). This area is interpreted as the histological ossification center; i.e., the area in which ossification of the hyaline cartilage begins and the growth record is most complete. The ossification center in *Andrias* spp. is very restricted (less than a 1 mm) and not easy to meet during sampling. Thus, the ossification center was not always met during sampling and the petrographic thin section originates sometimes from a locality somewhat distally or proximally to the exact ossification center (Fig. 1; Table 1). It is important to note that in humeri and femora of *Andrias* spp., the ossification center is slightly proximal to the smallest circumference of the morphological midshaft (i.e. where midshaft is narrowest) (Fig. 1). We did not analyze the micro-Ct-data any further. All our histological observations are based on petrographic thin sections of the area around the ossification center. Petrographic thin sections were prepared following the method described in Klein and Sander [36]. Preservation method (freezing vs.10% formalin storage vs. ethanol storage) had no appreciable influence on the histological structure visible in petrographic thin sections.

Osteohistological analysis was performed with a Leica DM LP polarizing light microscope, the pictures were obtained with a Leica DFC 420 equipped with an EOS Canon camera. The size of the medullary cavity was measured along the ventral to dorsal diameter. The histological nomenclature follows Francillon-Vieillot et al. [20] and de Buffrénil et al. [5].

Osteocyte lacunae are noticeably large throughout *Andrias* spp. tissue. To test whether there is a real size difference in osteocyte lacunae compared to other Lissamphibia, we measured osteocyte lacunae and conducted a two-sample t-test assuming unequal variances for two samples. Sample 1 is the average osteocyte lacunae size from *A. japonic*us and *A. cf. davidianus* and sample 2 includes the average osteocyte lacunae sizes from other lissamphibians used for comparison (see Supplementary Information).

## Results

### Osteohistological description

#### Shape of cross section, nutrient canal, medullary cavity, and erosion

##### Andrias japonicus

**Fig. 2:**
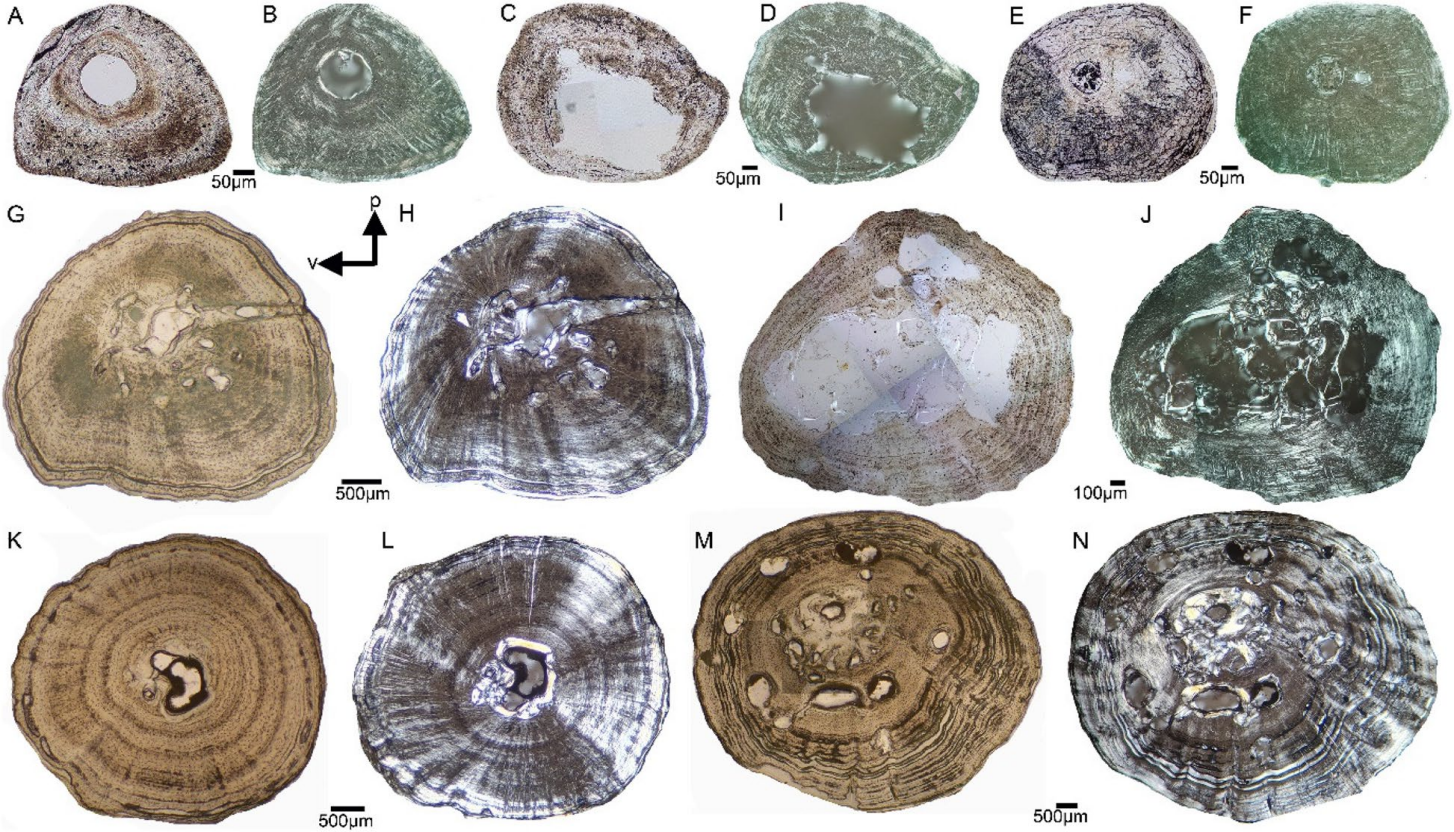
Humeral cross sections of *Andrias japonicus*. **A**, cross section of humerus of ASAGS-0078 in normal and **B**, polarized light. **C**, coss section of humerus of ASAGS-0077 in normal and **D**, polarized light. **E**, cross section of humerus of ASAGS-0050 in normal and **F**, polarized light. **G,** cross section of humerus of ASAGS-0100 in normal and **H**, polarized light. **I,** cross section of humerus of ASAGS-0197 in normal and **J**, polarized light. **K,** cross section of humerus of ASAGS-0106 in normal and **L**, polarized light. **M,** cross section of humerus of ASAGS-0101 in normal and **N**, polarized light. The shape of the cross section does not change sizeable. Most important is the change from a small central medullary cavity in small individuals (**A-F**) towards a central cavity that is accompanied (**G-H**; **K-L**) or even obscured by erosion cavities and sometimes by the deposition of (secondary) endosteal bone (**I-J**, **M-N**) in large individuals. Please note that the large size of the cavity in ASAGS-0197 (**I-J**) is a technical artefact. The presence of erosion cavities at the margin or close to the medullary cavity indicates the onset and increase of resorption activities. Secondary deposition of endosteal bone occurs in *Andrias* *japonicus* only at the margins of the erosion cavities and no cavity is completely filled. Note the differences in the amount of resorption and redeposition within the group of larger individuals (**G-H**, **K-L** versus **I-J**, **M-N**). A cyclicity in bone growth is visible in form of an alignment in rows of osteocytes in ASAGS-0197 (**I-J**) and in ASAGS-0106 (**K-L**). True LAGs (lines of arrested growth) are only identified in the outer cortex of ASAGS-0100 (**G-H**) and ASAGS-0106 (**K-L**) and throughout most of the cortex in ASAGS-0101 (**M-N**). Cyclicity in bone growth is best visible at the anterodorsal bone side, where the cortex is thickest. Humerus ASAGS-0050 (**E-F**) shows the nutrient canal as a round cavity/foramen to the right of the somewhat larger central medullary cavity. Humerus ASAGS-0100 (**G-H**) shows the nutrient canal as a real canal extending from the inner central medullary cavity to the outer cortex. In the other humeral cross sections, the nutrient canal is not visible. Most differences visible here are related to ontogeny. However, a certain intraspecific plasticity is also obvious. For details on age, size and sex of individuals see Table 1. Abbreviations: p-posterior, v-ventral

**Fig. 3:**
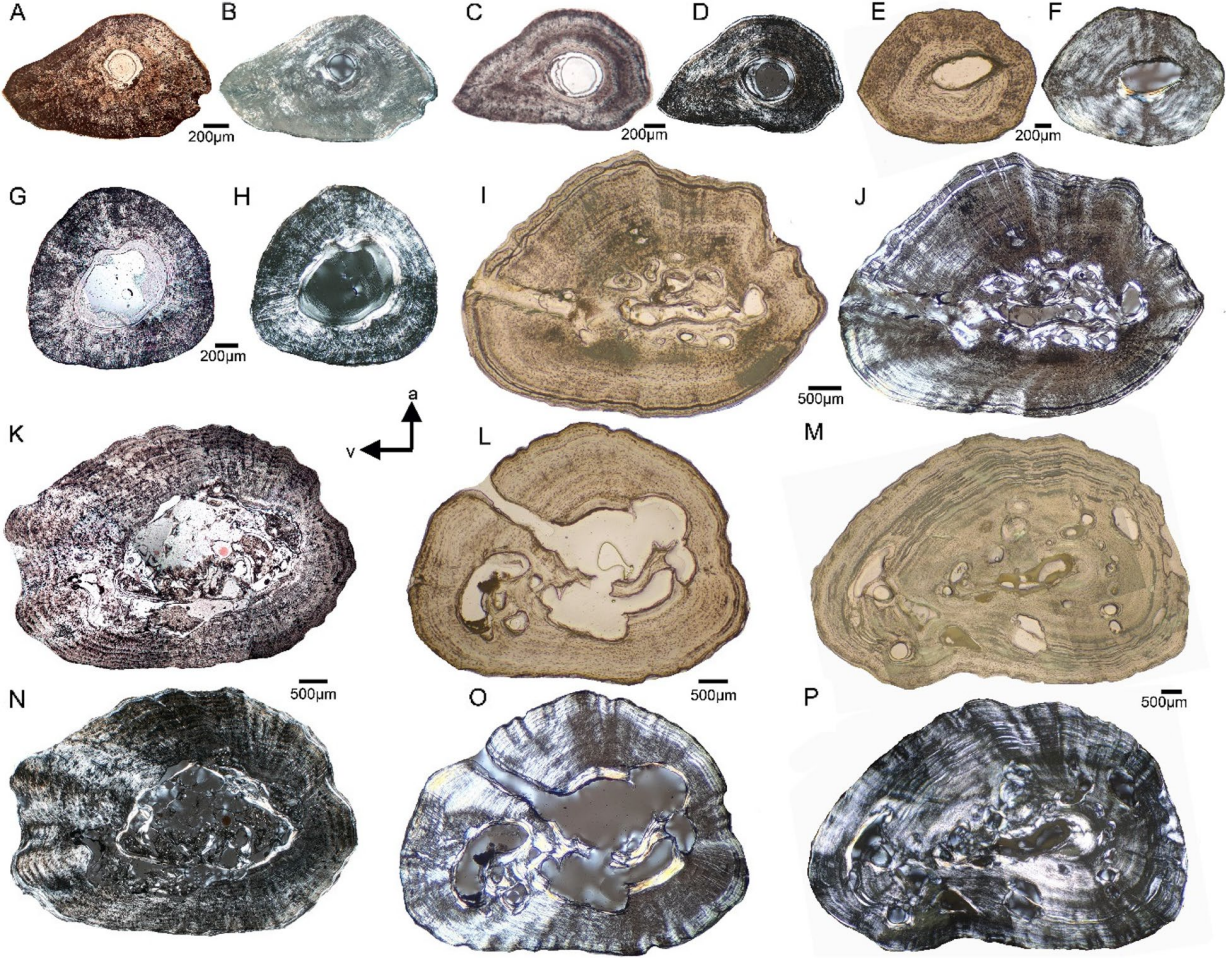
Femoral cross sections of *Andrias japonicus*. **A** cross section of femur of ASAGS-0078 in normal and **B**, polarized light. **C,** cross section of femur of ASAGS-0077 in normal and **D**, polarized light. **E**, cross section of femur of ASAGS-0317 in normal and **F**, polarized light. **G**, cross section of femur of ASAGS-0050 in normal and **H**, polarized light. **I**, cross section of femur of ASAGS-0100 in normal and **J**, polarized light. **K**, cross section of femur of ASAGS-0197 in normal and **N**, polarized light. **L**, cross section of femur of ASAGS-0106 in normal and **O**, polarized light. **M**, cross section of femur of ASAGS-0101 in normal and **P**, polarized light. The shape of the femoral cross sections is more variable than those of the humeri. The pointed (drop-shaped) end, that is well pronounced in very small individuals (**A-D**), represents the *linea aspera *region, which points ventroposteriorly in the living animal. A nutrient canal extending from the inner central medullary cavity to the outer cortex is visible in femur ASAGS-0100 (**I-J**) and ASAGS-0106 (**L**, **O**). In the other samples, the nutrient canal is not visible. Similar as in the humeri, a change from a small (**A-B**) or medium to large sized (**C-H**) central medullary cavity in small individuals towards a cavity that is accompanied (**I-J**, **M**, **P**) or even obscured by erosion cavities and the deposition of (secondary) endosteal bone (**K**, **N**; **L**, **O**) is visible. This indicates the onset and increase of resorption activities. Secondary deposition of endosteal bone occurs only at the margins of erosion cavities. Secondary bone deposition is however, more pronounced in ASAGS-0100 (**J**) and ASAGS-0101 (**P**). Note the differences within the group of larger individuals (**I-J**; **M**, **P** versus **K**, **N**, **L**, **O**). A cyclicity in bone growth in form of an alignment in rows of osteocytes is observed in the larger individuals ASAGS-0100 (**I-J**), ASAGS-0197 (**K**, **N**), and ASAGS-0106 (**L**, **O**), but except for the largest individual ASAGS-0101 (**M**, **P**). True LAGs (lines of arrested growth) are identified in the outer cortex of ASAGS-0100 (**I-J**) and ASAGS-0106 (**K-L**) and throughout most of the cortex of ASAGS-0101 (**M**, **P**). As in the humeri, most differences visible here are related to ontogeny but a certain intraspecific plasticity is also obvious. For details on age, size and sex of individuals see Table 1

Humeral samples that are taken from the ossification center range in shape from nearly triangular to round and oval throughout ontogeny (Fig. 2). Small femora show a pointed ventral margin (teardrop-shaped in the two smallest individuals ASAGS-0078 and ASAGS-0077), representing the morphological expression of the *linea aspera* (Figs. 3A-F). In larger individuals, the cross section of the femur is more elliptical with a less pronounced *linea aspera* (Figs. 3I-P). This is further corroborated by a more rounded shaft in the morphology of larger *A. japonicus* femora (Fig. 1). The external cortex of large humeri and femora exhibits a wave-like surface, characterizing the outer cross section from ASAGS-0100 and larger individuals (Figs. 2G-N, 3I-P; Table 1). A wave-like outer cortex is however, more pronounced in femora than in humeri.

The appearance of the nutrient canal at the ossification center is highly variable, if it is visible at all. In some samples, the nutrient canal extends as a real canal perpendicular from the medullary cavity to the outer cortex (humerus and femur ASAGS-0100, Figs. 2G, 3I-J; femur ASAGS-0106, Figs. 3L, O; Table 1). In humerus ASAGS-0050, only a large round foramen is recognizable immediately adjacent to the medullary cavity (Figs. 2E). The other samples do not show a nutrient canal or foramen.

The smallest individuals (ASAGS-0078, ASAGS-0077 and ASAGS-0050; Figs. 2A-F, 3A-H; Table 1) show a small, round, and centrally located medullary cavity with no or only slight erosion at the margin of the cavity. Humerus ASAGS-0050 exhibits a small medullary region filled by chondrocytes and calcified cartilage (Figs. 2E-F, 5I). The medullary cavity and region are lined by a thin but distinct and closed (i.e. running all around) layer of endosteal lamellar bone (Figs. 2B, F, 3B, D, F, H, 5A-C, I, K). In the femur of ASAGS-0050, the medullary cavity is larger (> 50% of cross-sectional diameter; Table 1) and the layer of endosteal bone is relatively thicker when compared to the other samples (Figs. 3G-H). In addition, its cross section is round. In general, the medullary cavity tends to be larger in femora than in humeri (Figs. 2, 3; Table 1). Kastschenko’s line, which represents the remains of the former cartilaginous shaft and indicates the retention of the complete sequence of periosteal bone deposition [13, 31], is clearly visible in the humerus and femur of the smallest individual (ASAGS-0078), and in the femur of ASAGS-0077 (Fig. 5B), the second smallest individual. It might have been present in the humerus of ASAGS-0077 as well but here the central region of the cross section was damaged during preparation (Figs. 2C-D). Kastschenko’s line, is also faintly visible in humerus and femur of ASAGS-0050 and femur of ASAGS-0317.

The larger specimens ASAGS-0100, ASAGS-0197 and ASAGS-0101 (Table 1), show distinct erosion of the inner periosteal cortex by large erosion cavities (Figs. 2G, 3I-P, 5D-F). The erosion process has destroyed the shape and lining of the medullary cavity (Figs. 2G-J. M-N, 3I-P; 5D-F; Table 1). In ASAGS-0100 (Figs. 2G, 3I-J, 5F) and ASAGS-0101 (Figs. 2 M-N, 3M, P, 5E), distinct amounts of secondary endosteal lamellar bone have filled some of the erosional rooms. The amount of secondary endosteal deposits is less in the humerus and femur of ASAGS-0197, which is also corroborated by the highest degree of erosion relative to the remaining samples (Figs. 2I, 3 K, N). The large erosional rooms are here traversed by secondary trabeculae with large erosion bays and a cartilaginous matrix. In femur ASAGS-0106, erosion is also intensive (Figs. 3L, O; Table 1). However, in the humerus of ASAGS-0106 erosion is lowest when compared to other larger samples (Figs. 2 K-L, 5D). Here, the closed layer of endosteal bone surrounding the medullary cavity is still present and intact (Fig. 5D), which is reminiscent of ontogenetically younger stages such as in the humerus of ASAGS-0078 and ASAGS-0050 (Figs. 2A–B, E–F, 5A, I).

##### *Andrias* cf. *davidianus*

**Fig. 4:**
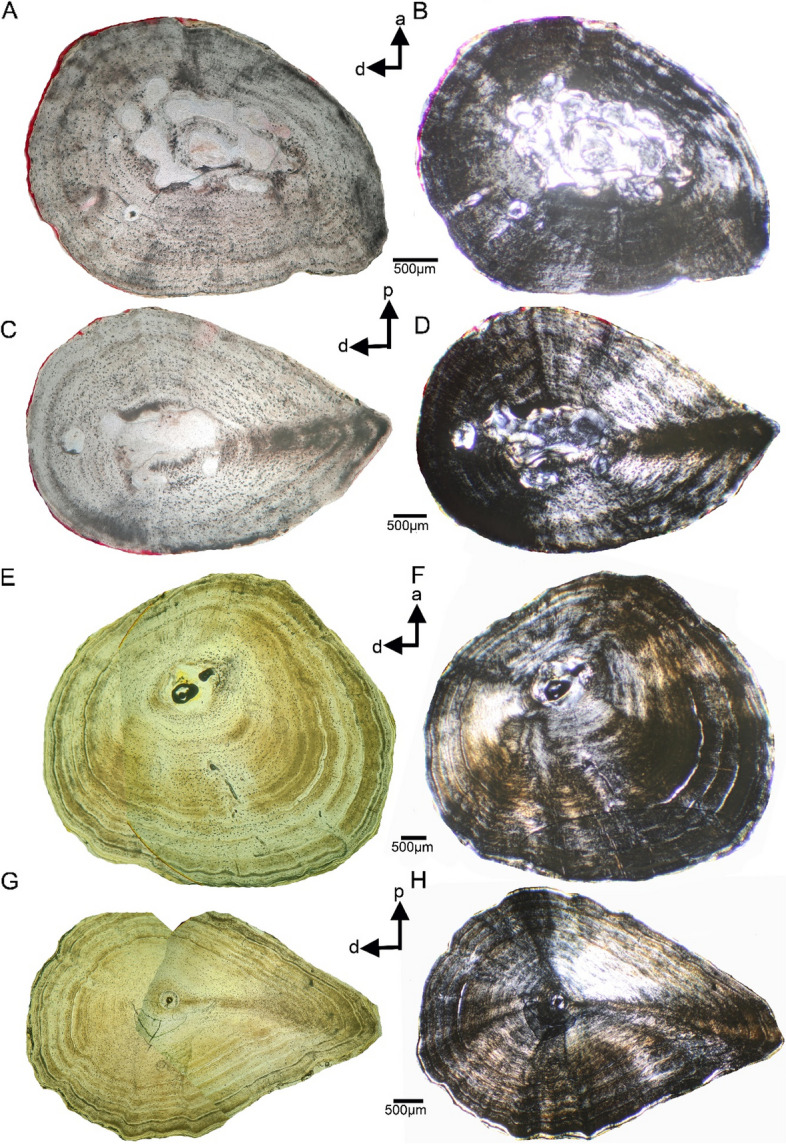
Humeral and femoral cross sections of *Andrias *cf. *davidianus*. **A** cross section of humerus of ZFKM 8568 in normal and **B**, polarized light. **C**, cross section of femur of ZFKM 8568 in normal and **D**, polarized light. **E**, cross section of humerus of ZFKM 97391 in normal and **F**, polarized light. **G**, cross section of femur of ZFKM 97391 in normal and **H**, polarized light. Please note the differences between the shape of the cross sections of the two humeri (**A-B**, **E-F**) of the two individuals whereas the femora of both individuals are both drop-shaped (**C-D**, **G-H**). Femur ZFKM 8568 (**C-D**) shows the nutrient canal as a round cavity/foramen to the left of the medullary region (**C**, **D**). In both humeri (**E**, **F**), a spur of the nutrient canal is in form of aligned roundish and radial narrow cavities indicated. These cavities are not embedded into the matrix as would be typical for periosteal vascular canals and do not show any further characteristics of those. They are thus not interpreted as part of a periosteal vasculary system. Humerus and femur of ZFKM 8568 (**A-D**), both show no clear medullary cavity but erosion cavities and some secondary endosteal bone redeposition, indicating resorption activities (**B**, **C**). Humerus and femur of ZFKM 97391 (**E-H**) do not show any significant resorption or remodeling. The very small, if not reduced, medullary cavity (F, humerus) and medullary region (H, femur) are unique among the here studied *Andrias* spp. sample. Please see Fig. 5G (humerus) and 5H (femur) for an enlargement of each of the two endosteal regions. Cyclicity in bone growth in form of an alignment in rows of osteocytes is observed in both, ZFKM 8568 (**A-D**) and ZFKM 97391 (**E-H**). True LAGs (lines of arrested growth) occur only throughout the cortex of both elements of ZFKM 97391. For details on age, size and sex of individuals see Table 1

The humerus and femur of ZFMK 8568, both show a drop-shaped cross section, with the femoral one being more pronounced (Figs. 4A). The humerus of ZFMK 97391 is round (Figs. E–F). The femur exhibits again a pronounced drop-shaped cross section (Figs. 4G). The outer surface is wave-like in the femur of ZFMK 97391 (Figs. 4G). The wave-like surface is only slightly visible in the humerus of ZFMK 97391 (Figs. 4E) and in the femur of ZFMK 8568 (Figs. 4C). A wave-like outer surface is absent in the humerus of the latter (Figs. 4A). In ZFMK 8586, the nutrient canal is visible as a round foramen at the dorsal bone side (Figs. 4B, D) and in the figured micro-Ct-picture (Fig. 1B4). The humerus of ZFMK 97391 shows some aligned radial to round openings (Fig. 4E), potentially representing traces of the marginally cut nutrient canal.

**Fig. 5:**
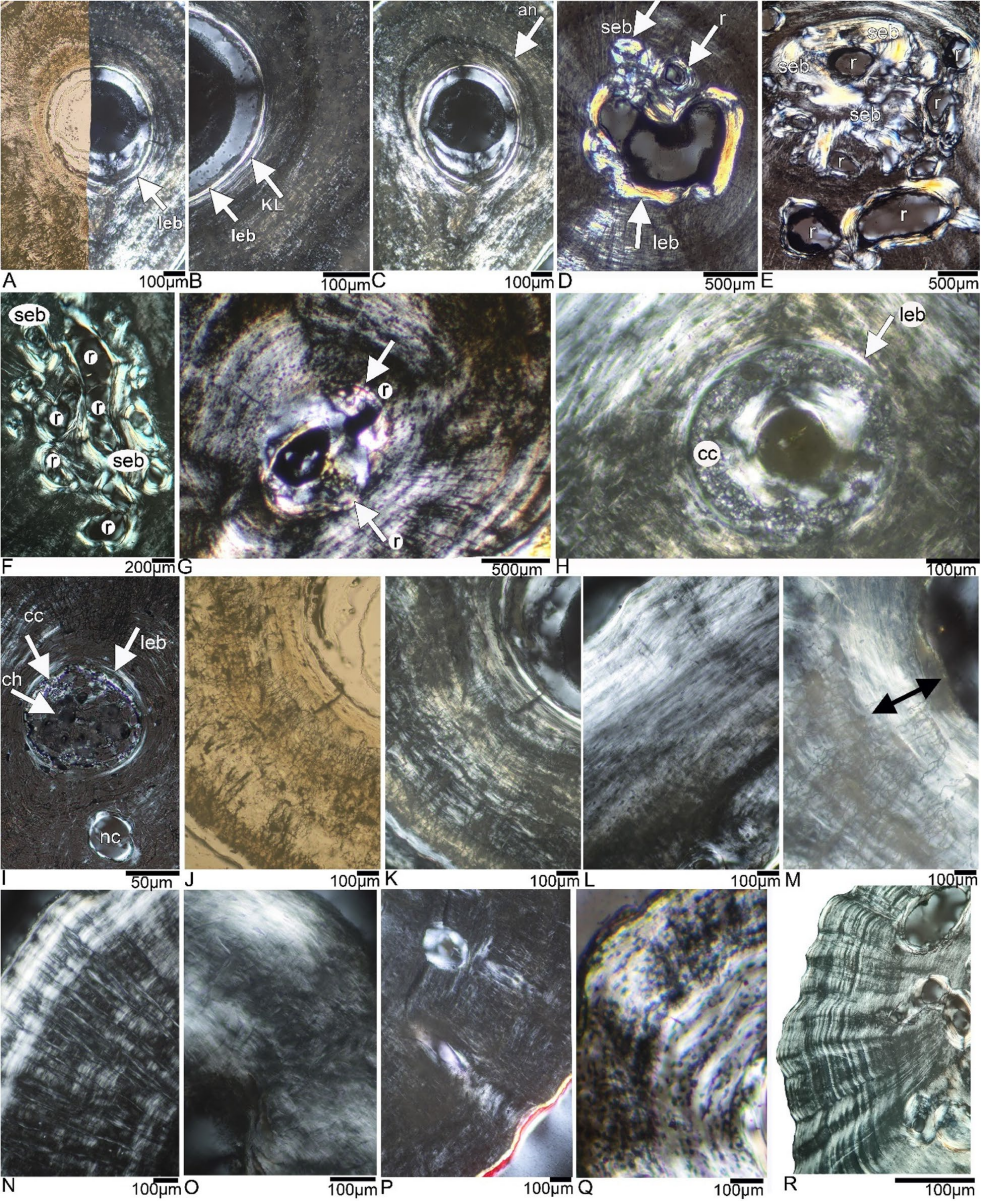
Histological details of *Andrias *spp. tissue. **A**, lining of the medullary cavity by a layer of endosteal bone in femur ASAGS-0078 in normal and polarized light. **B**, lining of the medullary cavity by a layer of endosteal bone and a well visible Kastschenkos’s line in femur ASAGS-0077 in polarized light. **C**, distinct, but only partially preserved annulus, separating an inner tissue, which started highly organized and grades then into loosely organized and coarse parallel-fibered tissue in femur ASAGS-0078 in polarized light. **D**, thick layer of endosteal bone (yellowish) around the enlarged medullary cavity as well as some resorption and infilling of an erosion cavity by secondary endosteal bone in humerus ASAGS-0106 in polarized light. **E**, medullary region showing large resorption (i.e. erosion cavities) as well as resorbed areas already infilled by secondary endosteal bone in humerus ASAGS-0101 in polarized light. **F**, medullary region showing equal resorption and deposition of secondary endosteal bone in femur ASAGS-0100 in polarized light. **G**, tiny medullary cavity surrounded by minor resorption in humerus ZFMK 97391 in polarized light. Note the distinct Sharpey’s fibers in the right lower corner (black stripes). **H**, a tiny medullary cavity surrounded by a layer of calcified cartilage in femur ZFMK 97931 in polarized light. **I**, medullary region surrounded by a layer of endosteal bone and filled by local accumulations of calcified cartilage and chondrocytes (black dots) in humerus ASAGS-0050 in polarized light. **J**, coarse parallel-fibred tissue in the femur ASAGS-0077 in normal and **K**, polarized light. Note also the dense network of canaliculi and large osteocyte lacunae (both black). **L**, from the inner to the outer cortex: increase in organization in already highly organized parallel-fibred tissue in the cortex of femur ASAGS-0317 in polarized light. **M**, highly organized tissue (double headed arrow) in the innermost cortex of femur ASAGS-0078 in polarized light. Note also the dense network of canaliculi. **N**, scares (i.e. having left holes in the tissue) of Sharpey’s fibers in humerus ASAGS-0050 in polarized light.** O**, coarse (dark areas) and highly organized (bright areas) parallel-fibred tissue intermixed in the femur ASAGS-078 in polarized light. **P**, “holes” in loosely organized parallel-fibred tissue in humerus ZFMK 8568 either representing vascular canals (what we render unlikely due to the lack of characteristics of periosteal vascular canals, see text) or the marginally cut nutrient canal in polarized light. **Q**, diffuse annuli (bright thin areas) in femur ASAGS-0317 in polarized light. **R**, distinct LAGs (lines of arrested growth) in the outer cortex of humerus ASAGS-0101 in polarized light. Abbreviations: an, annulus; cc, calcified cartilage; ch, chondrocytes; leb, layer of endosteal bone; Kl, Kastschenkos’s line; r, resorption; seb, eb, secondary endosteal bone

The humerus of ZFMK 8568 shows intensive erosion as well as deposits of secondary endosteal bone (Figs. 4A). Erosion and redeposition is also visible in the femur of the same individual but both is less pronounced (Figs. 4C–D). The medullary cavity of humerus ZFMK 97391 is extremely small (Figs. 4E–F, 5G). The femur has a small medullary region filled with calcified cartilage (Figs. 4G–H, 5H). The medullary region of the femur is lined by a thin layer of endosteal bone (Figs. 4F, H, 5H). No erosion is visible in the femur (Figs. 4G–H, 5H). In the humerus, minor erosion seems to have taken place at the margin of the medullary cavity (Figs. 4E–F, 5G).

## Tissue and vascularity in *Andrias* spp.

All samples of *Andrias* spp. share a principally parallel-fibred tissue. Mainly in the smaller individuals, the fiber arrangement of the parallel-fibred tissue is very coarse and/or loosely organized, which is evident from dimly lit birefringent regions (Figs. 2, 3, 4, 5). During stage rotation, different areas of the section span from highest to lowest birefringence and vice versa. When the section displays a consistently lowly lit appearance, the tissue blocks much of the incoming light, suggesting that the matrix of the tissue is unorganized i.e., the collagen bundles are distributed in multiple directions. In larger individuals, the dimly lit coarse parallel-fibered tissue is gradually being replaced by a more brightly lit higher organized parallel-fibered tissue.

The entirety of each section is very dimly lit in ASAGS-0078 (Figs. 2B, 3B, 5A, C, M), ASAGS-0077 (Figs. 2D, 3D, 5B, J, K), and ASAGS-0050, indicating a coarse parallel fibered tissue. Tissue organization clearly tends to increase in these small individuals towards the outer surface, except for ASAGS-0050 which does not exhibit any highly organized periosteal tissue (Figs. 2F, 3H). The humerus of ASAGS-0050 exhibits the highest amount of this coarse tissue (Fig. 2F, 5I).

The fiber arrangement is very coarse in femur ASAGS-0101, but intermixed with regions that are better organized (Figs. 3 M, P; Table 1). In other larger individuals, the parallel-fibred tissue is highly organized (ASAGS-0317, ASAGS-0100, ASAGS-0106; ZFMK 97931) partially even grading into lamellar bone (Figs. 2, 3, 4). This highly organized parallel fibered bone is mostly circumferential and periosteal in its distribution corroborated by extremely bright birefringence towards the periphery. Unlike the other ontogenetically younger individuals, the small femur of ASAGS-0317 exhibits no coarse parallel-fibred tissue (Figs. 3F, 5L). Femur ASAGS-0197 (Figs. 3K, N) displays a brightly lit high organized parallel-fibered tissue, however, the periosteal margin of the sample is lowly lit (= coarse parallel-fibred tissue). ZFMK 8568 has loosely organized parallel-fibred tissue i.e., it is neither very coarse nor highly organized (Figs. 4B, D, 5P), whereas both samples of ZFMK 97931 show highly organized parallel-fibered tissue (Figs. 4F, H, 5H).

Beside traces of the nutrient canal in some samples (Table 1), there are neither any unequivocal simple vascular canals nor any primary osteons in the midshaft area of any sample, indicating no development of primary vascularization. The visible openings in humerus of ZFMK 8568 and of ZFMK 97391, are interpreted to represent traces of the marginally cut nutrient canal (Figs. 4B, E, 5P). They do not show any feature of primary simple vascular canals or primary osteons, i.e., no smooth enclosing of the vessel into the matrix and no surrounding bone lamellae [20:209]. All other cavities in the periosteal cortex are identified as erosion cavities due to their actively eroding margin and/or secondary filling of endosteal bone or preparatory artefacts (i.e., holes).

Osteocyte lacunae are numerous in all samples (Figs. 2, 3, 4, 5, specimens shown in normal light, 5A, J). They are very large and round in all individuals of *A. japonicus* (Figs. 2, 3, 5A, J; Table 1; Supplementary information Fig. 1). However, in femora of small ASAGS-0078 and ASAGS-0077 (Figs. 3A-D) and both elements of ASAGS-0106 (Figs. 2 K-L, 3L, O), the osteocyte lacunae are less numerous and flatter, although still larger when compared to other Lissamphibia (see Supplementary information). The change in osteocyte lacunae shape from round to flat and in arrangement is usually related to an increase in tissue organization. Flat osteocyte lacunae distribution aligned in rows (= dynamic osteogenesis; see [18]) is characteristic of highly organized parallel-fibred and lamellar tissue, whereas round and scattered osteocyte lacunae (= static osteogenesis; see [18]) are typical of the coarse parallel-fibered tissue. Independent of the skeletal element, tissue, and ontogenetic age, osteocyte lacunae are very large in *Andrias japonicus* and *A.* cf. *davidianus*. Statistical tests revealed that the average size of osteocyte lacunae in *Andrias* spp. is significantly larger than in any other sampled lissamphibians (see Supplementary information).

Accompanied with the coarse parallel-fibred tissue is a dense network of thick canaliculi (Figs. 2, 3, 4, 5A, J, M; Supplementary information Fig. 1) connecting the osteocyte lacunae and/or each other. In samples from larger individuals, this network is often collapsed, and broken parts of the smaller/thinner canaliculi are scattered across the entire cortex. However, some larger samples do show local accumulations of extremely thick and dense canaliculi.

## Growth record

In small individuals of *A. japonicus* (ASAGS-0078, ASAGS-0077, ASAGS-0050; Figs. 2A-F; Fig. 3A-H) and in ZFMK 8568 (*A.* cf. *davidianus*; Figs. 4A-D), growth marks occur in form of thin and diffuse annuli (Fig. 5Q). These are thin layers of highly organized avascular parallel fibered tissue (Fig. 5Q) having a similar birefringence as the brightly lit endosteal lamellar band. However, these diffuse annuli are sometimes difficult to discern around the entire cross section either due to poor development or to their being obscured by the coarse nature of parallel-fibred tissue or by the highly organized parallel-fibred tissue, both of which can mask the separation between annuli and zones due to bright birefringence (Figs. 2, 3, 4, 5Q).

In larger individuals of *A. japonicus* (ASAGS-0100, ASAGS-0197, ASAGS-0101, Figs. 2G-N, 3I-J, M-P) and *A.* cf. *davidianus* (ZFMK 97391; Figs. 4E-H), a transition from diffuse annuli in the inner cortex to distinct lines of arrested growth (LAGs; thin, but distinct opaque rest lines; Fig. 5R) in the outer cortex is observed. The change from annuli to LAGs is accompanied by a tendency (but not always strict or regular) toward increased tissue organization.

Cyclicity in growth is also indicated by an alignment of osteocyte lacunae in rows parallel to the surface and by changes in tissue organization (Fig. 5L). In the smaller individuals (ASAGS-0078, ASAGS-0077, ASAGS-0050; Figs. 2A–F, 3A–H) as well as in some of the larger ones (humerus ASAGS-0100, Figs. 2G–H; femur ASAGS-0101, Figs. 3 M-P; both samples of ZFMK 97391, Figs. 4E–H), the innermost cortex is made of a highly organized avascular parallel-fibred tissue that is well delimited by a change in tissue and by an annulus (Fig. 5C).

## Sharpey’s fibers

The cross sections of all individuals of *A. japonicus*, independent of ontogenetic stage contain abundant Sharpey’s fibers (SF) (Figs. 2, 3, 5N). These are very distinct, extending from the inner cortex to the outer surface, i.e. usually perpendicular to the bone surface (Figs. 2, 3, 5N). They are always present in the pointed ventral tip (*linea aspera* region) of the femora (Fig. 3, 5N). Humeri of smaller individuals exhibit a criss-crossing (i.e., perpendicular intersection of SF coming from two different directions) of SF (Figs. 2B, D, F; 5G, N), which is not visible in the larger individuals (Figs. 2G–N). Comparatively, in the femora of ASAGS-0078 and ASAGS-0077, the density of SF is extremely high at the region of the *linea aspera* (Figs. 3B, D). Humeri exhibit a more uniform and lower amount of SF. In smaller individuals of the *A. japonicus* samples, the SF are not yet mineralized and left only a perforation in the tissue that appear as empty radial strips (Figs. 5N).

Except for the ventral femoral bone side, fibers also occur usually all around the other bone sides, at least in a single distinct bundle but often several bundles are in parallel distributed all over the cross section. At the posterior bone side, fibers are usually less common. Sometimes the fibers are shorter, not reaching from the inner to the outer cortex but restricted to parts of the cortex. The coarse nature of the parallel-fibred tissue sometimes resembles, or is indeed intermixed, with short fibers (Figs. 2, 3, 5). There is a certain disparity in SF expression along the ontogenetic series of stylopod bones of *A. japonicus* (Figs. 2, 3). The two *A.* cf. *davidianus* individuals display a lower amount of SF, even in the *linea aspera* region (Figs.4D, H).

## Discussion

### Nutrient canal, endosteal tissue, erosion, and microanatomy

#### *A. japonicus*

The appearance of the nutrient canal in the stylopod elements of *Andrias* spp. is prone to high plasticity. Although all samples in the present study were taken at or near the histological ossification center, the nutrient canal is sometimes fully exposed, sometimes only marginally cut (i.e., round foramen), or even not cut. Thus, the nutrient canal seems not always to run strictly perpendicular between medullary cavity and outer bone surface at the ossification center. There are not many studies dealing with the location of the nutrient canal. However, Houssaye and Prevoteau ([29] and references therein) have also documented a highly intraspecific and intraskeletal variation in the position of the nutrient canal in mammals.

Small individuals show a small medullary cavity, which is slightly larger in femora than in humeri (Table 1), and which is lined by a layer of endosteal bone. Larger individuals show a medullary region, which is the result of resorption of inner periosteal tissue and the expansion of the endosteal region. Resorption varies in degree in the different individuals, but with a tendency to being more pronounced in femora than in humeri (Table 1). In some of the larger individuals, redeposition of endosteal bone had occurred in erosional rooms. Thus, there is a general shift during ontogeny from osteosclerotic stylopodial elements in juvenile individuals to larger individuals that show relative bone mass decrease but in a highly variable degree.

#### *A. *cf. *davidianus*

The position of the nutrient canals is similar variable in the two ZFMK samples. ZFMK 8568 shows a similar degree of erosion and redeposition when compared to *A. japonicus*. The larger individual (ZFMK 97931), however, is unique among the current *Andrias* spp. sample, as it shows a small medullary cavity lined by a layer of endosteal bone. Despite its large size (94 cm; Table 1), ZFMK 97931 shows no (humerus) or nearly no (femur) resorption and redeposition. The nearly nonexistent medullary cavities of the stylopod bone elements of ZFMK 97391 renders them osteosclerotic and thus, in this respect, comparable to smaller individuals of *A. japonicus*. The presence of calcified cartilage in the femur sample of ZFMK 97391 is notable. Only one other sample, the humerus of the small *A. japonicus* individual ASAGS-0050, shows a small medullary region filled by calcified cartilage. However, the latter was not sampled exactly at the ossification center and it is due to its size (28.5 cm) and age (8.6 yrs), a sexually immature individual. No other *Andrias* spp. samples show any remains of calcified cartilage at midshaft at any ontogenetic stage. The presence of calcified cartilage in an *A. davidianus* individual is interpreted as being neotenic in nature by Canoville et al. [12]. Because only one of the adult specimens in our sample has calcified cartilage preserved at the ossification center, we conclude that the presence of calcified cartilage in an adult individual is exceptional. The presence of calcified cartilage in ZFMK 97391 may be related to general variability in the rate of cartilage resorption in different individuals and along the shaft or the femur was a long-ago regenerated element [32]. However, both possibilities have to be tested in a future study by analyzing the distribution of cartilage along the shaft and by studying unequivocally regenerated elements.

## Presence of a closed layer of endosteal bone surrounding the medullary cavity

A closed layer of endosteal bone around the medullary cavity is in *Andrias japonicus* only observed in humeri and femora of small individuals (Table 1). In larger individuals, this layer of endosteal bone is largely destroyed by active resorption of the periosteal cortex and the transformation of the medullary cavity into a medullary region. The latter is also visible in ZFMK 8568. ZFMK 97391 lacks any resorption. The femur of ZFMK 97391 has its tiny medullary cavity surrounded by a closed layer of endosteal bone, which is, however, surrounded by a layer of calcified cartilage. The humerus shows only remains of a layer of endosteal bone around the medullary cavity. In other available studies of Lissamphibia, the medullary cavity is usually surrounded by a layer of endosteal bone (e.g., [12, 58, 59, 64, 62], whereas Temnospondyli show not a layer of endosteal bone (i.e., [66, 41] (Teschner et al. 2018 [69]) but maybe the samples of Temnospondyli studied to date were already too old or too large.

The pattern of erosion and redeposition by secondary endosteal bone seems also to differ when compared to other lissamphibians (e.g., [3, 12, 13]) and requires further in-depth comparison and analysis.

## Coarse parallel-fibred tissue

Parallel-fibred tissue is usually faster deposited than lamellar bone due to a lower degree of organization of the parallel-fibred tissue, but it is not as rapidly deposited and loosely organized as woven bone (e.g., [5–8, 20]). Parallel-fibred tissue is thus interpreted as indicating a medium growth rate. However, organization and density of vascularization is an important factor that highly influences growth rates. The loosely organized and coarse parallel-fibred tissue observed in *Andrias* spp. may indicate a faster growth rate in *Andrias* when compared to other Lissamphibia, but without further studies on the deposition rates of avascular (see below), loosely organized and coarse parallel-fibred tissue, we are unable to make any statement about growth rate in *Andrias* spp. However, given the long-time larvae of *Andrias japonicus* need to grow until they reach active reproduction (in average about 15 yrs at an average size of about 60 cm; see method section and Table 1), large body sizes in *Andrias* spp. were likely reached by an extension of ontogeny rather than by an increase in growth rate, as is the case in other cryptobranchids [64]. An extended ontogeny may be also indicated by the observation that faster growth to a certain body size in captive individuals does not correspond to earlier onset of reproductive activities [27].

According to Castanet et al. [13], there is a clear relationship in Lissamphibia between tissue complexity and body-size, which is however not well understood. Several reasons are discussed such as ontogenetic factors (rate of growth), biomechanical demands, and longevity. Factors controlling the precise segregation of bony tissues appear to be mostly "functional" in the broad sense [13].

All modern Lissamphibia grew with highly organized parallel-fibred or lamellar tissue [12, 13]. Stem salamander and a cryptobranchid from the Lower Cretaceous of Uzbekistan show well organized parallel-fibred or lamellar tissue as well (e.g., [63–65]. Some large Triassic temnospondyls show loosely organized (but not coarse) parallel-fibred tissue (e.g., [40, 41]) but this tissue is always moderately to well vascularized and neither coarse nor avascular as in *Andrias*. Coarse, but well vascularized, parallel-fibred tissue is so far mainly described in Triassic marine reptiles (summarized in [37]). Thus, the presence of avascular, loosely organized, and coarse parallel-fibred tissue in *Andrias* spp. is unique among Lissamphibia (and other tetrapods). This tissue seems not to be related to an increased growth rate (due to the lack of vascularization) in *Andrias* spp. However, it may be related to the large body size of *Andrias* spp. and presumably may point to structural constraints of forming large bones solely by (avascular) highly organized tissue (i.e. highly organized parallel-fibred and lamellar tissue) in large sized taxa. However, this is quite hypothetical and requires further study.

## The lack of vascularization in *Andrias* spp.

Parallel-fibred tissue can be avascular as in some extant Lissamphibia [12], in small monitor lizards [4] and other small squamates ([62]; pers. obs NK). However, the above-mentioned examples are all small (< 460 mm body length i.e., [4]) and grew with well to highly organized parallel-fibred tissue.

The avascular nature of the parallel-fibred tissue around the ossification center in petrographic thin sections in our samples of *Andrias* spp., differs from descriptions from other studies about *Andrias*. Sanchez et al. [60:Suppl.7], Canoville et al. [12] and Buffrénil et al. ([6: Fig. 4.9A]) described simple vascular canals for *Andrias* spp. on the basis of petrographic thin sections, micro- or synchrotron-Ct-scans. Skutschas et al. [64] reconstructed a network of cavities in a femur of *Cryptobranchus* sp. by micro-Ct-data. Thus, the presence or absence of primary vascular canals may also be prone to plasticity, as is evident for other histological features of *Andrias* spp. Modern Lissamphibia show usually—but not uniformly—avascular tissue [3, 12]. Bones of large Temnospondyli are usually well vascularized (e.g., [40, 41]).

The dense network of canaliculi permeating the otherwise avascular cortex in our *Andrias* spp. sample (Supplementary Information Fig. 1) is striking. Canoville et al. [12] described canaliculi as poorly developed in their sample of *Andrias japonicus*, which may be related to the later ontogenetic stage of their sample. Similar dense networks of canaliculi as we observed in our sample of *Andrias* spp., have been figured but are not explicitly mentioned in other Lissamphibia. Castanet et al. [13] mentioned that the osteocyte lacunae in the zones of Lissamphiba form “a more extensive canalicular system”. The cortex of a humerus of a modern fire salamander figured in Organ et al. (56: Fig. 1A) shows large osteocyte lacunae with an intensive network of canaliculi. The anuran *Trichobatrachus robustus* has to our sample of *Andrias* spp. comparably long, branching canaliculi in the femoral cortex [12]. Canoville et al. [12] further wrote that the cross-sections of most anuran limb bones reveal the presence of complex canalicular networks in the vicinity of the osteocyte bodies and that canaliculi are also present in the layer of lamellar endosteal bone [12:117] as is the case in our sample of *Andrias japonicus*. *Bombina orientalis* also shows dense networks of canaliculi ([62:Suppl.Plate1-4]). Skutschas and Stein [63] mentioned that osteocyte lacunae in the periosteal cortex of *Kokartus* are numerous and interconnected by thin numerous canaliculi.

Canoville et al. [12] pointed out that when bone cortices are very thin, the capillaries of the periosteum and osteocyte canaliculi can supply the bone whereas in thicker cortices, a vascular network is necessary. However, exceptions to this common knowledge are known [4] and additional factors must influence vascularity [12]. Lai et al. ([48:1]) stated that “osteocytes form an interconnected network in the lacunar-canalicular pore system buried within the mineralized matrix which allows osteocytes to obtain nutrients from the blood supply”.

Thus, due to the lack of vascular canals in our sample of *Andrias* spp., we hypothesize the possibility that the intense network of canaliculi observed in *Andrias* spp. may have assumed the function of vascular canals and supplied the tissue. In larger specimens, the cortex is thicker and the erosion cavities scattering here the inner periosteal cortex can host a vascular system that supports the metabolic functions of the bone. However, this hypothesis needs further study and testing. The reasons for the failure to develop vascular canals must be examined in future studies.

## Size of osteocyte lacunae

A statistical test revealed that osteocyte lacunae of *Andrias* spp., throughout ontogeny are in average relative larger when compared to other modern lissamphibians (Supplementary information). Already Castanet et al. [13] documented large osteocyte lacunae in caudates. This was corroborated by a study of Organ et al. [56] that measured osteocyte lacunae size to link it with genome size. Our sample of *Andrias* spp. now documents an even larger osteocyte lacunae size for *Andrias* spp. when compared to other Caudata.

The lack of vascular canals may be an important factor that favors the increase of the size of osteoblasts and later osteocytes, as part of a hypothetical nutritional network (see above). Osteocytes are highly active cells, which are indispensable for the normal function of the skeleton, playing major roles in several physiological processes [48, 72].

However, this is only a preliminary result, and we refrain from going into detail here. A more detailed study including a larger comparable sample and a refined methodical approach is necessary to conduct a detailed analysis and allow for further quantitative and qualitative assertions.

## Sharpey’s fiber expression across *Andrias* samples

Mainly in the smaller samples of *A. japonicus*, Sharpey’s fiber** (**SF) being extrinsic non-mineralized fibers, which is also documented for some frogs [39]. This is interpreted as functioning to retain a higher level of flexibility compared to the fibers of the underlying bone tissue. However, these stripes are not to be confused with simple vascular canals,they are much too thick and irregular in shape. In addition, circular polarized light nicely visualizes the true nature of these radial stripes as Sharpey’s fibers ([30] in press). Castanet et al. [13] stated that extraneous collagenous fibers from the periosteum, or of tendinous or ligamentous origin, once engulfed in the bone matrix ultimately become Sharpey's fibers (or extrinsic bone fibers) common in periosteal and dermal bone [13:1638]).

*Andrias japonicus* shows in general a higher expression of Sharpey’s fiber (SF) in humeri and mainly in the femora than *A.* cf. *davidianus* does. The disparity in SF expression in the femora and humeri of small and large *A. japonicus* individuals indicates that SF formation varies also significantly during ontogeny, suggesting a variable biomechanical loading on the shafts of stylopod elements of *A. japonicus* during growth. Thus, differences in the amount of SF between *A. japonicus* and *A.* cf. *davidianus* may be related to general biomechanical differences in the attachment of tendon and/or muscles in the two different taxa, or may be related to ontogeny. Different housing condition of the captive individuals may have contributed to differences in SF as well.

Alternatively, SF visibility may be affected by the underlying bone tissue. Since the predominant tissue in the younger samples of *A. japonicus* is coarse parallel fibered bone, SF penetration is easier to identify due to low mechanical compactness of the coarse bone tissue, resulting in higher SF expression in terms of density and distribution. Thus, in the tissue of older/larger individuals, such as those ZFMK samples where tissue is higher organized, the visibility of SF may be less pronounced.

Deep attachment of soft tissues, i.e. SF, is usually identified as muscles connected to bones indirectly through a tendon [57] or as tendinous or indirect muscle insertion and ligamentous attachments [67], or fibrocartilage holding a tendon towards the outer surface of bone [39]. However, there is very little documentation of SF from any skeletal element of modern salamanders. So far studied fossil stem-salamanders do not show SF [63, 65]. SF are well known to be preserved in the long bone fossils of temnospondyls [41, 40, 42, 69, 75]. The obvious absence of SF in the admittedly small sample of stem- and modern salamanders (except for *Andrias* spp.) may be related to the tendency toward miniaturization in most modern salamanders. Besides body size, a stronger biomechanical load on stylopod bones in *Andrias* spp. is also conceivable. However, because the sample of *A. japonicus* studied here was kept in tanks, there may be also a relation to environmental conditions.

## Preliminary insights to the growth pattern of *Andrias* spp.

The innermost cortex of the smallest samples (ASAGS-0078 and ASAGS-0077) is made of a highly organized avascular parallel-fibred tissue that is well delimited by the change in tissue and by a distinct growth mark (i.e. annulus) from the coarse or loosely organized parallel-fibered tissue visible in later stages (Table 1). This tissue is interpreted as the tissue of the earliest ontogenetic periosteal growth stage and it might indicate the phase during which yolk was absorbed before the individual starts feeding. According to Niwelinski [54], this phase is temperature depending and can last between 30 and 80 days. In the larger individuals (Table 1), the inner cortex is made of a similarly slow deposited tissue resembling the dimension of the cross sections of ASAGS-0078 and ASAGS-0077 (Fig. 5C, I). In any case, the initial phase of growth was slower than the following phases.

The age and size when first reproduction start is highly variable in *Andrias* spp. [2, 68, 73]; pers. obs. Asa Zoo team). According to body-size data from wild populations [47, 43, 44] compared to observations made in Asa Zoo, captive females seem to start reproductive activity at a larger size than in the wild. If they mature roughly at the same age is not known due to the lack of age data from wild populations. In nearly all tetrapods, the onset of sexual maturity (i.e. active reproduction) is accompanied by a decrease in growth rate (i.e. change in tissue and vascularization), and often also by a change in the sequence and/or manifestation of growth marks due to the lower growth rate (summarized in [5] and references therein). In large Temnospondyli, some taxa develop LAGs also only late in ontogeny [66], producing in earlier ontogenetic stages a sequence of annuli and zones [41].

Such an increase in tissue organization accompanied by a switch from diffuse annuli to well-developed lines of arrested growth (LAGs) is visible in the petrographic thin sections of the larger individuals of the current sample of *Andrias* spp. (Table 1). Thus, the occurrence of distinct LAGs in petrographic thin sections appears to be an indicator for actively reproducing individuals in *Andrias* spp. Female ASAGS-0100 (14 yrs, 73.5 cm) is the youngest individual that shows such an increase in tissue organization and one very clear LAG in its outermost cortex. However, male ASAGS-0197 (19yrs, 70 cm) does not show any clear LAGs, but does show an increase in tissue organization in its outermost cortex. We conclude that both individuals were already active reproducing or close to. None of the smaller and younger individuals of *A. japonicus* shows any distinct LAGs in the petrographic thin sections (Table 1).

Both elements of ASAGS-0100 and ASAGS-0197 show intensive erosion. The bones of ASAGS-0100 display additionally deposits of secondary endosteal tissue. None of the smaller and younger individuals presents any erosion or redeposition of bone. It thus seems that with active reproduction or maybe with reaching a certain body size, erosion in the stylopod elements of *Andrias japonicus* starts. This is accompanied by the loss of a well-delimited medullary cavity and a decrease in bone mass.

ZFMK 8568 (*A.*cf. *davidianus*) shows distinct erosion and diffuse annuli, but no distinct LAGs have been yet deposited nor is an increase in tissue organization documented. Thus, from the current knowledge and interpretation, the individual was not yet actively reproducing (at a body size of 62 cm). Housing conditions are unknown, but likely the individual was well fed and had grown rapidly but was not yet sexually mature despite its large size. The constant temperature in the housing conditions may have subsequently prevented the deposition of distinct LAGs.

ZFMK 97391 was a sexually mature, advanced adult, as is indicated by numerous distinct LAGs in the outer half of its cortex and an increase in tissue organization, although no erosion is visible.

## Notes for future skeletochronological studies

Yamasaki et al. [80] found LAGs in phalanges of an 11-year-old (55 cm) and younger individuals. In our sample, none of the smaller individuals show distinct LAGs in the petrographic thin sections. This discrepancy can be explained by the fact that Yamasaki et al. [80] used microtomic/biological thin sections. Petrographic and microtomic sections reveal different information’s and have different optical features [62].

The study of Yamasaki et al. [80] undoubtedly documented that skeletochronology in microtomic sections of phalanges is a reliable method to assess age in *Andrias japonicus*.

However, no samples from individuals older than 11 years were included in this former study and thus no actively reproducing individuals or any older and larger sized individuals had been studied. However, growth data from the latter group are essential to understand the general growth pattern and life history traits of *Andrias*. Data representing the complete ontogenetic series are also necessary to allow for mathematical growth modeling and deducing allometries (e.g., [24] and references therein). Thus, additional studies on the growth record of *Andrias* spp. are important and are in progress. The status of *Andrias* spp. as an endangered species, their long lifespans, and the difficulty of conducting long-term monitoring studies render the individuals from the Asa Zoo vital for skeletochronological and osteohistological studies. The preliminary results of the current study and the less clear growth marks in early ontogenetic stages in our petrographic thin sections show that, for future skeletochronological studies of *Andrias*, microtomic sections are preferable to petrographic thin sections.

## Conclusions

*Andrias* spp. grows with an avascular, coarse and loosely organized parallel-fibred tissue that later in ontogeny becomes more organized. The avascular and coarse nature of the tissue of *Andrias* spp. is unique among Lissamphibia but also among other tetrapods. The avascularity in *Andrias* spp. is likely related to a phylogenetic constraint within modern Lissamphibia.

The avascular tissue of *Andrias* spp. is permeated by a dense network of canaliculi that might have in combination with the enlarged osteocyte lacunae nourished the bone instead of vascular canals. Osteocyte lacunae size is in average statistically relatively larger throughout ontogeny in *Andrias* spp.

For the determination of reproductive active individuals, the visible transition from diffuse annuli to distinct LAGs in petrographic thin sections, accompanied by an increase in tissue organization, is important. Although not within the scope of the current study, we find that for skeletochronological studies petrographic thin sections are less suited because at least in early ontogenetic stages growth marks are not very clear and difficult to count.

Besides a tendency toward increased tissue organization and the change from annuli to LAGs, further observed ontogenetic changes are related to the transition from a small, well delimited medullary cavity to a medullary region by the onset and increase of resorption in larger individuals. Some large individuals also show secondary filling of the erosional cavities by endosteal bone.

The osteohistology (e.g., tissue, avascularity, osteocyte lacunae size, network of canaliculi, resorption and redeposition pattern) of *Andrias* spp. is complex and unique among modern Lissamphibia as well as when compared to so far studied fossil taxa. The lack of comparable material of Lissamphibia however, hampers the interpretation of histological features observed in *Andrias* spp. Although our sample size is limited, high developmental plasticity for all histological features is obvious in *Andrias* spp.

Although our sample is based on zoo animals and thus does not reflect natural conditions (i.e., typical osteohistology of wild individuals), our results are nonetheless important in the light of developmental plasticity, osteohistological features, and the detection of a possible histological marker for the onset of sexual maturity in giant salamanders. Our results will further assist to the osteohistological study and interpretation of histological features of large extinct amphibians (Temnospondyli).

## Supplementary Information

Supplementary Material 1.

## Data Availability

The datasets used and/or analyzed during the current study are available from the corresponding author on reasonable request and will be finally stored in the Hiroshima City Asa Zoological Park.
