# Supplementary material for: Unique bone histology of modern giant salamanders: a study on humeri and femora of Andrias spp
Source: Zoological Lett. 2024 Oct 18;10:18. doi: 10.1186/s40851-024-00240-1 (PMC11488364; doi:10.1186/s40851-024-00240-1)
Supplement: Supplementary file 1 — Supplementary Material 1. [file 40851_2024_240_MOESM1_ESM.pdf]

**Supplemental Material I-III**

## I. Row data: Measurements of osteocyte lacunae size in *Andrias japinicus, Andrias davidianus* and complementary Lissamphibia.

To reveal an average size estimate for osteocyte lacunae size osteocyte lacunae were measured in each humerus and femur thin section (Suppl. Tabs 1-3). From each cross section, an area (camera field view) from the periosteal cortex was chosen that shows a high density of well-preserved osteocyte lacunae (Suppl. Figs. 1-2). Between 10 and 20 osteocyte lacunae had been measured in each thin section (Suppl. Tabs 1-3). Of each osteocyte lacuna, the longest exposed diagonal length was measured but if was not focused through.

As complementary data, the same procedure was carried out for six additional taxa: Anura (*Bombina bombina*, *Rana temporaria*, *Lithobates catesbeianus*) and Caudata (*Ichthyosaura alpestris alpestris*, *Salamandra salamandra*, *Ambystoma mexicanum*)

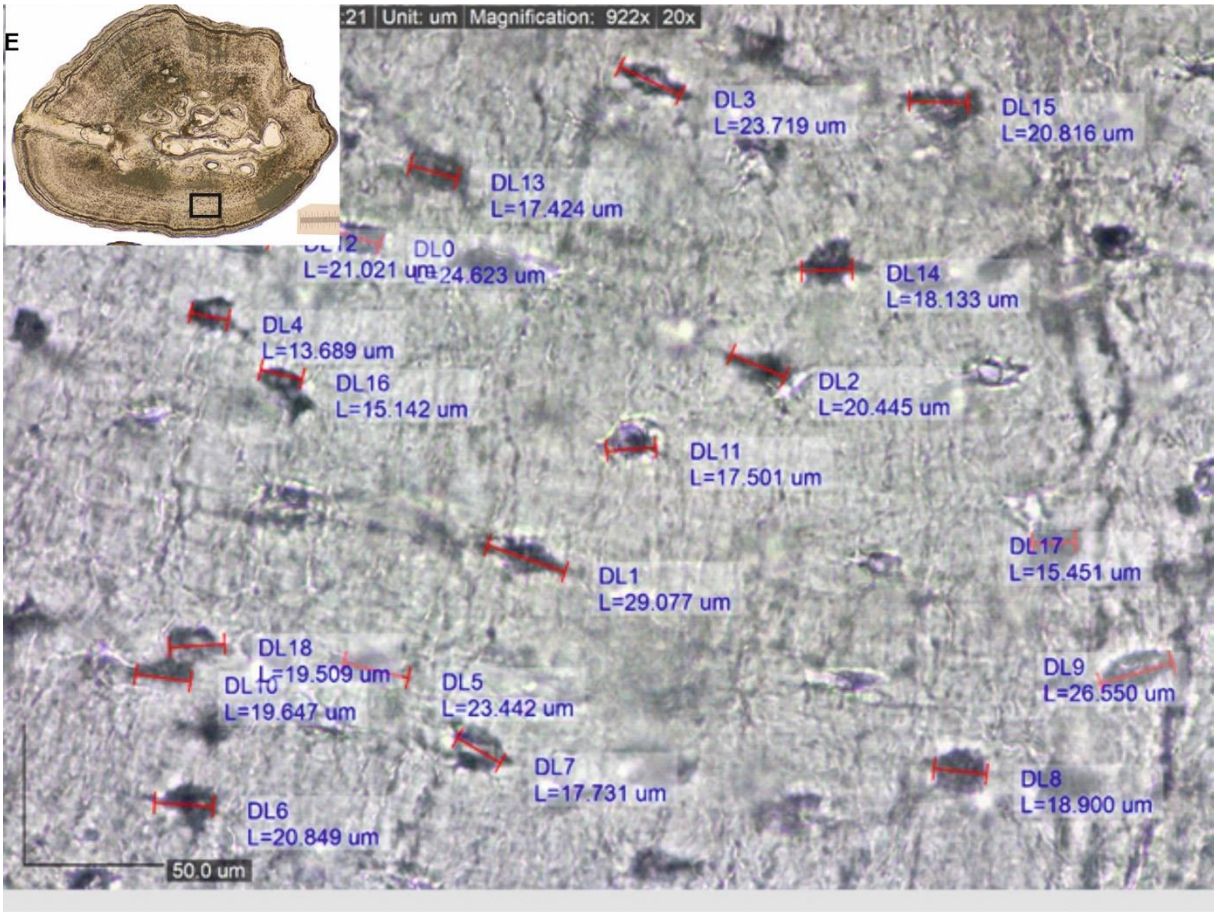


**Suppl. Fig. 1**. Osteocyte measurements using the femur of *Andrias japonicus* AGAGS-0100 as an example. Note that the focus in this case is on the osteocytes, not the tissues.

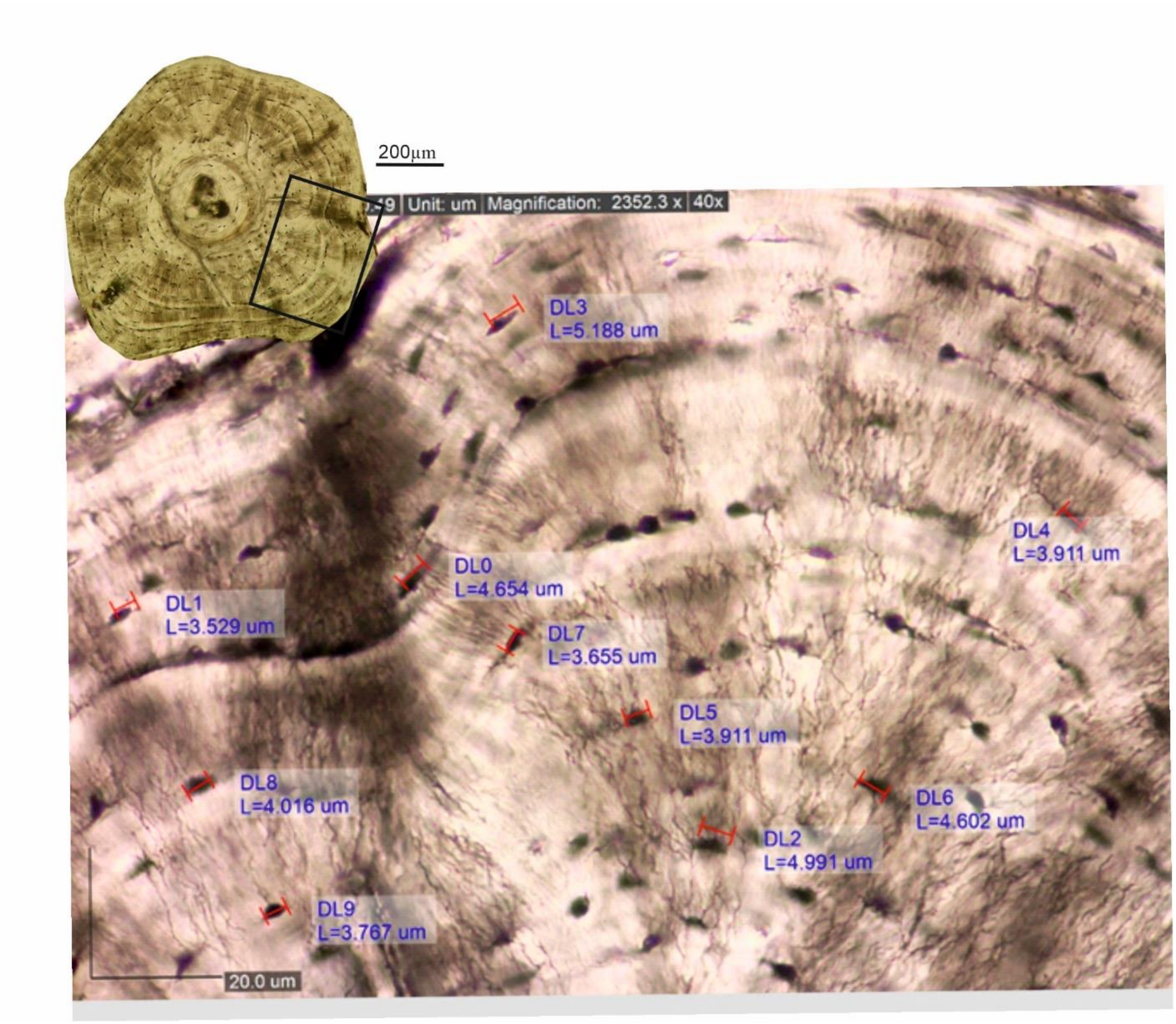


**Suppl. Fig.2**. Osteocyte measurements using the femur of *Bombina bombina* as an example. Note that the focus in this case is on the osteocytes, not the tissues.

**Suppl. Tab. 1.** Measurement of osteocytes maximum length (in µm) among *Andrias japonicus*.

| AGAGS-0078 | | AGAGS-0077 | | AGAGS-0317 | | AGAGS-0050 | | AGAGS-0100 | | AGAGS-0197 | | AGAGS-0106 | | AGAGS-0101 | |
|---|---|---|---|---|---|---|---|---|---|---|---|---|---|---|---|
| h | f | h | f | h | f | h | f | h | f | h | f | h | f | h | f |
| 19,819 | 17,111 | 23,297 | 14,78 | | 18,257 | 8,082 | 20,903 | 10,843 | 21,021 | 15,953 | 16,779 | 18,133 | 22,819 | 25,583 | 21,021 |
| 9,609 | 20,838 | 20,179 | 19,567 | | 12,099 | 13,322 | 13,579 | 14,221 | 17,424 | 11,409 | 30,916 | 15,61 | 17,45 | 26,854 | 28,929 |
| 15,319 | 13,689 | 20,489 | 26,474 | | 8,109 | 14,658 | 12,006 | 25,503 | 24,623 | 16,163 | 28,695 | 19,475 | 20,816 | 27,525 | 22,907 |
| 10,925 | 14,269 | 24,941 | 19,219 | | 14,158 | 14,826 | 15,186 | 23,728 | 13,689 | 17,908 | 28,695 | 16,33 | 22,561 | 31,629 | 22,819 |

| | | | | | | | | | | | | | | | |
|---|---|---|---|---|---|---|---|---|---|---|---|---|---|---|---|
| 14,11 | 18,121 | 22,967 | 16,576 | | 17,255 | 17,501 | 15,494 | 12,822 | 15,142 | 19,475 | 32,804 | 17,833 | 19,739 | 26,414 | 32,25 |
| 14,12 | 14,519 | 22,561 | 14,826 | | 9,396 | 10,822 | 15,567 | 18,133 | 19,509 | 15,91 | 30,387 | 12,321 | 21,644 | 25,273 | 25,157 |
| 12,155 | 10,006 | 20,729 | 15,654 | | 17,255 | 15,142 | 16,899 | 14,094 | 19,647 | | 35,323 | 13,44 | 21,737 | 26,482 | 19,83 |
| 10,843 | 19,089 | 20,412 | 20,379 | | 12,91 | 26,879 | 24,198 | 23,728 | 20,849 | | 31,091 | 16,792 | 28,828 | 24,393 | 21,644 |
| 14,363 | 18,121 | 24,43 | 18,319 | | 20,145 | 13,44 | 17,463 | 19,819 | 29,077 | | 26,482 | 16,819 | 19,567 | 16,33 | 24,614 |
| 17,46 | 16,899 | 18,648 | 18,514 | | 10,759 | 17,565 | 12,752 | 14,094 | 23,442 | | 25,697 | 13,031 | 23,49 | | 19,567 |
| 18,331 | 14,269 | 26,209 | 9,679 | | 23,576 | 10,822 | 15,567 | 12,822 | 17,731 | | | 21,518 | 20,19 | | 16,779 |
| 11,586 | 12,892 | | 14,902 | | 12,734 | 14,094 | 12,769 | 10,759 | 23,719 | | | 22,799 | 22,137 | | 24,254 |
| 17,871 | 17,411 | | 20,719 | | 16,233 | 10,822 | 16,121 | 16,603 | 18,133 | | | 26,98 | 20,719 | | 17,424 |
| | 15,953 | | 14,237 | | 15,711 | 17,203 | 12,91 | | 20,445 | | | 15,91 | 18,17 | | 17,111 |
| | 17,655 | | | | | | | | 17,501 | | | 17,411 | 23,49 | | 26,55 |
| | 22,31 | | | | | | | | 20,816 | | | 20,19 | | | 27,377 |
| | 18,84 | | | | | | | | 15,451 | | | 21,064 | | | 15,007 |
| | 17,501 | | | | | | | | 26,55 | | | 13,031 | | | 18,071 |
| | 14,11 | | | | | | | | 18,9 | | | | | | 21,487 |
| | 13,59 | | | | | | | | | | | | | | 19,647 |
| | 18,121 | | | | | | | | | | | | | | 21,664 |
| **14,35** | **16,44** | **22,26** | **17,42** | | **14,90** | **14,66** | **15,82** | **16,71** | **20,19** | **16,14** | **28,69** | **17,70** | **21,56** | **25,61** | **22,78** |

**Suppl. Tab. 2.** Measurement of the osteocytes' maximum length (in µm) among *Andrias davidianus.*

| ZFMK97391 | | ZFMK8568 | |
|---|---|---|---|
| humerus | femur | humerus | femur |
| 11,429 | 14,735 | 19,006 | 21,685 |
| 22,158 | 24,198 | 15,319 | 18,071 |
| 29,439 | 17,45 | 15,007 | 20,903 |
| 19,034 | 14,519 | 17,463 | 24,254 |
| 16,992 | 13 | 18,563 | 21,984 |
| 20,967 | 13,228 | 13,506 | 15,91 |

| | | | |
|---|---|---|---|
| 15,436 | 11,25 | 14,363 | 20,675 |
| 15,436 | 13,868 | 18,429 | 17,45 |
| 18,071 | 13,771 | 17,871 | 17,424 |
| 22,957 | 13,59 | 18,563 | 16,979 |
| 29,962 | 14,856 | 16,33 | 19,75 |
| 21,01 | 17,111 | 11,488 | 21,685 |
| 22,501 | 15,91 | 11,17 | 16,385 |
| 17,908 | 12,91 | 13,966 | 15,007 |
| 18,986 | | 18,17 | 13,372 |
| 15 | | 18,804 | |
| | | 14,719 | |
| | | 15,725 | |
| **19,83** | **15,06** | **16,03** | **18,77** |

**Suppl. Tab. 3. Measurement of the osteocytes' maximum length (in µm) among selected taxa of modern Anura and Urodela.**

| *Ichthyosaura alpestris alpestris* | | *Ambystoma mexicanum* | *Bombina bombina* | | *Rana temporaria* | *Lithobates catesbeianus* | *Salamandra salamandra* |
|---|---|---|---|---|---|---|---|
| humerus | femur | femur | humerus | femur | femur | femur | humerus |
| 4,743 | 3,43 | 6,582 | 4,836 | 5,118 | 3,83 | 5,423 | 2,999 |
| 6,577 | 4,988 | 7,427 | 5,026 | 3,529 | 3,683 | 3,664 | 2,482 |
| 4,526 | 3,2 | 6,99 | 4,991 | 4,654 | 2,941 | 5,168 | 3,179 |
| 4,684 | 3,946 | 7,91 | 4,684 | 3,655 | 2,591 | 4,594 | 2,746 |
| 6,534 | 3,911 | 5,268 | 7,533 | 4,016 | 3,079 | 3,51 | 2,708 |
| 5,398 | 5,493 | 5,811 | 4,378 | 3,767 | 3,254 | 4,495 | 1,86 |
| 4,242 | 4,109 | 7,224 | 6,885 | 3,911 | 2,833 | 3,51 | 2,105 |
| 5,294 | 3,664 | 7,441 | 6,738 | 3,911 | 3,157 | 5,679 | 2,055 |
| | 5,549 | 7,108 | 4,118 | 4,991 | 2,941 | 5,58 | 2,353 |
| | 4,007 | | 4,109 | 4,602 | 2,248 | 4,743 | 2,353 |
| | 5,242 | | | | 4,084 | 5,168 | 3,168 |
| | 4,594 | | | | | 4,495 | 2,906 |
| | | | | | | 4,48 | 2,746 |
| | | | | | | 5,493 | 1,86 |
| | | | | | | 3,51 | 2,708 |

| | | | | | | | |
|---|---|---|---|---|---|---|---|
| | | | | | | 5,549 | 3,655 |
| | | | | | | 6,789 | 2,631 |
| | | | | | | 5,06 | |
| **5,25** | **4,34** | **6,86** | **5,33** | **4,21** | **3,15** | **4,83** | **2,62** |

**Suppl. Tab. 4.** Measurement of osteocytes maximum length (in µm) in long bones of *Andrias* spp. and other taxa of Lissamphibia.

| specimen number | stylopod | average individual osteocytes size for bone | cumulative average osteocytes size for specimen |
|---|---|---|---|
| AGAGS-0078 | humerus | 14,35 | 15,40 |
| | femur | 16,44 | |
| AGAGS-0077 | humerus | 22,26 | 19,84 |
| | femur | 17,42 | |
| AGAGS-0317 | humerus | - | 14,90 |
| | femur | 14,90 | |
| AGAGS-0050 | humerus | 14,66 | 15,24 |
| | femur | 15,82 | |
| AGAGS-0100 | humerus | 16,71 | 18,45 |
| | femur | 20,19 | |
| AGAGS-0197 | humerus | 16,14 | 22,41 |
| | femur | 28,69 | |
| AGAGS-0106 | humerus | 17,70 | 19,63 |
| | femur | 21,56 | |
| AGAGS-0101 | humerus | 25,61 | 24,20 |
| | femur | 22,78 | |
| ZFMK97391 | humerus | 19,83 | 17,45 |
| | femur | 15,06 | |
| ZFMK8568 | humerus | 16,03 | 17,40 |
| | femur | 18,77 | |
| *Ichthyosaura alpestris alpestris* | humerus | 5,25 | 4,80 |
| | femur | 4,34 | |
| *Ambystoma mexicanum* | humerus | - | 6,86 |
| | femur | 6,86 | |
| *Bombina bombina* | humerus | 5,33 | 4,77 |
| | femur | 4,22 | |
| *Rana temporaria* | humerus | - | 3,15 |
| | femur | 3,15 | |
| *Lithobates catesbeianus* | humerus | - | 4,83 |
| | femur | 4,83 | |
| *Salamandra salamandra* | humerus | 2,62 | 2,62 |
| | femur | - | |

**II. Finding statistical increase of osteocyte lacunae size in giant salamanders relative to other lissamphibians.**

A “two sample t-test assuming unequal variances for two samples” was conducted with the average osteocyte lacunae sizes. Sample 1 includes the average osteocyte lacunae size from the **ontogenetic series** of *A. japonicus* and adults of *A. davidianus*; sample 2 includes average osteocyte lacunae sizes from other **adult** lissamphibians used for comparison.

The test found a statistically significant difference in the size of osteocyte lacunae, i.e., the osteocyte lacunae size is significantly larger in *Andrias* spp. relative to the other sampled lissamphibians. Please note that this is only a preliminary study and test to document a in general larger osteocyte lacunae size in *Andrias* spp.

**Suppl. Tab. 5.** Average osteocyte lacunae size (in µm) in *Andrias* and other lissamphibians *sp*. includes data from the ontogenetic series of *A. japonicus*.

| **specimen number** | **cumulative average osteocytes size for specimen** |
|---|---|
| ASAGS-0078 | 15,40 |
| AGAGS-0077 | 19,84 |
| AGAGS-0317 | 14,90 |
| AGAGS-0050 | 15,24 |
| AGAGS-0100 | 18,45 |
| AGAGS-0197 | 22,41 |
| AGAGS-0106 | 19,63 |
| AGAGS-0101 | 24,20 |

| | |
|---|---|
| ZFMK97391 | 17,45 |
| ZFMK8568 | 17,40 |
| *Ichthyosaura alpestris alpestris* | 4,80 |
| *Ambystoma mexicanum* | 6,86 |
| *Bombina bombina* | 4,77 |
| *Rana temporaria* | 3,15 |
| *Lithobates catesbeianus* | 4,83 |
| *Salamandra salamandra* | 2,62 |

**Suppl. Tab. 6**. Results of two sample t test shows statistically significant difference in the osteocyte lacunae size increase in giant salamanders relative to other lissamphibians.

| | *Variable 1* | *Variable 2* |
|---|---|---|
| Mean | 18,4901706 | 4,504667 |
| Variance | 9,600780489 | 2,243145 |
| Observations | 10 | 6 |
| Hypothesized Mean Difference | 0 | |
| df | 14 | |
| t Stat | 12,10906738 | |
| P(T<=t) one-tail | 4,16351E-09 | |
| t Critical one-tail | 1,761310136 | |
| P(T<=t) two-tail | 8,32701E-09 | |
| t Critical two-tail | 2,144786688 | |

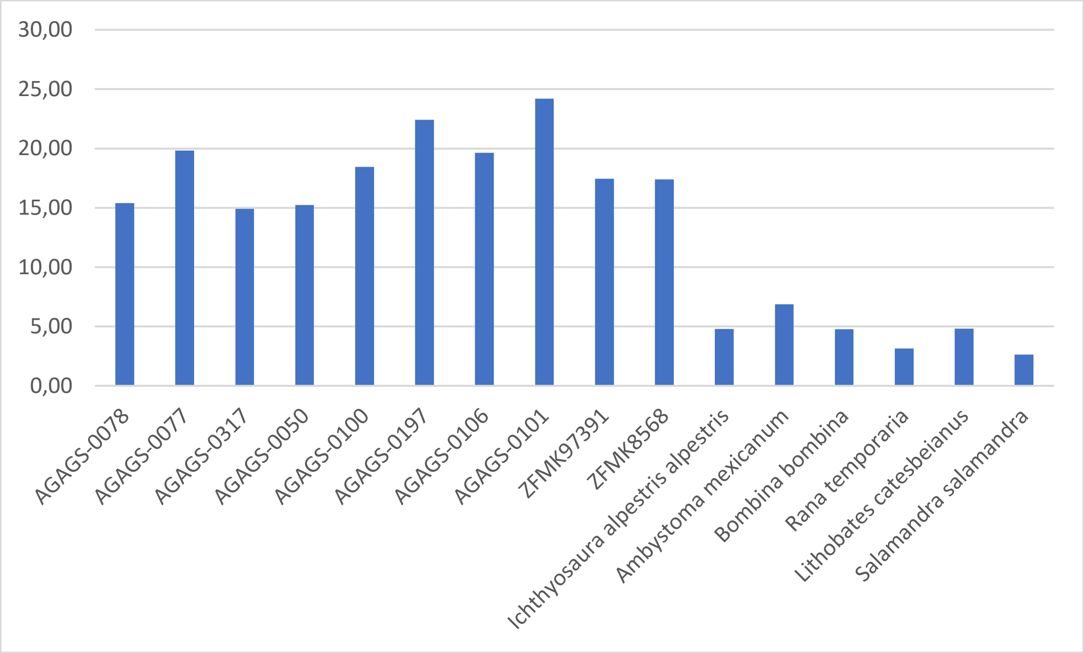


Graph 1 shows that all *Andrias* specimens sampled herein exhibit osteocytes larger than 10 micrometers which is not observed in the other lissamphibians.

A “two sample t-test assuming unequal variances for two samples” was conducted with the average osteocyte lacunae sizes. Sample 1 includes the average osteocyte lacunae size from the **adults** of *A. japonicus* and *A. davidianus*; sample 2 includes average osteocyte lacunae sizes from other **adult** lissamphibians used for comparison. This additional test was carried out to check if the sample size of osteocyte measurements from adult *Andrias* would show statistical significance when compared with the remaining lissamphibians sample.

The test found a statistically significant (**p=0.00000895517**) difference in the size of osteocyte lacunae, i.e., the osteocyte lacunae size is significantly larger in *Andrias* spp. relative to the other sampled lissamphibians.

**Suppl. Tab. 7.** Average osteocyte size (in µm) in *Andrias* and other lissamphibians *sp*. Includes data only from the adult *Andrias*.

| species (bones unspecified) | cumulative average osteocytes size for specimen |
|---|---|
| AGAGS-0197 | 22,41 |

| | |
|---|---|
| AGAGS-0106 | 19,63 |
| AGAGS-0101 | 24,20 |
| ZFMK97391 | 17,45 |
| ZFMK8568 | 17,40 |
| *Ichthyosaura alpestris alpestris* | 4,80 |
| *Ambystoma mexicanum* | 6,86 |
| *Bombina bombina* | 4,77 |
| *Rana temporaria* | 3,15 |
| *Lithobates catesbeianus* | 4,83 |
| *Salamandra salamandra* | 2,62 |

**Suppl. Tab. 8.** Results of two sample t test shows statistically significant difference in the osteocyte size increase in giant salamanders relative to other lissamphibians. Includes data only from the adult *Andrias*.

| | *Variable 1* | *Variable 2* |
|---|---|---|
| Mean | 20,21663648 | 4,504667 |
| Variance | 9,155085877 | 2,243145 |
| Observations | 5 | 6 |
| Hypothesized Mean Difference | 0 | |
| df | 6 | |
| t Stat | 10,58129147 | |
| P(T<=t) one-tail | 2,09648E-05 | |
| t Critical one-tail | 1,943180281 | |
| P(T<=t) two-tail | 4,19296E-05 | |
| t Critical two-tail | 2,446911851 | |

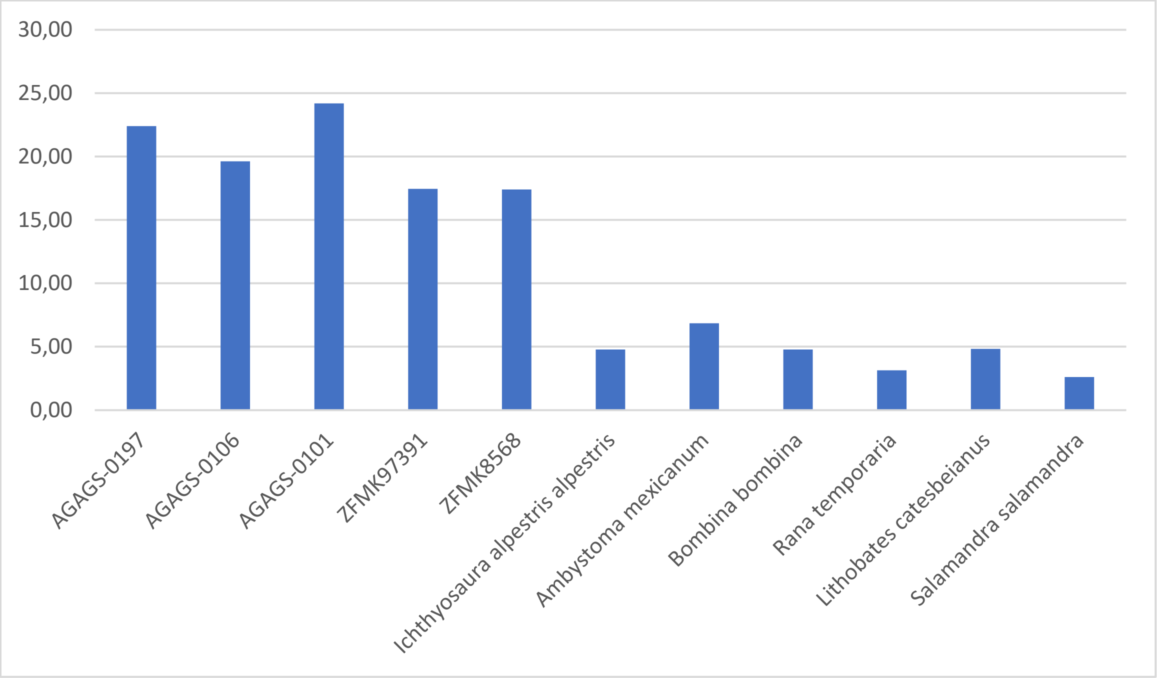


Graph 2 shows that adult *Andrias* specimens sampled herein exhibit osteocytes larger than 10 micrometers which is not observed in the other lissamphibians.
